# Breeding With Major and Minor Genes: Genomic Selection for Quantitative Disease Resistance

**DOI:** 10.3389/fpls.2021.713667

**Published:** 2021-08-06

**Authors:** Lance F. Merrick, Adrienne B. Burke, Xianming Chen, Arron H. Carter

**Affiliations:** ^1^Department of Crop and Soil Sciences, Washington State University, Pullman, WA, United States; ^2^United States Department of Agriculture-Agricultural Research Service Wheat Health, Genetics and Quality Research Unit, Department of Plant Pathology, Washington State University, Pullman, WA, United States

**Keywords:** genomic selection, fixed-effect, disease resistance, stripe rust, genome-wide associate studies, rrBLUP

## Abstract

Disease resistance in plants is mostly quantitative, with both major and minor genes controlling resistance. This research aimed to optimize genomic selection (GS) models for use in breeding programs that are needed to select both major and minor genes for resistance. In this study, stripe rust (*Puccinia striiformis* Westend. f. sp. *tritici* Erikss.) of wheat (*Triticum aestivum* L.) was used as a model for quantitative disease resistance. The quantitative nature of stripe rust is usually phenotyped with two disease traits, infection type (IT) and disease severity (SEV). We compared two types of training populations composed of 2,630 breeding lines (BLs) phenotyped in single-plot trials from 4 years (2016–2020) and 475 diversity panel (DP) lines from 4 years (2013–2016), both across two locations. We also compared the accuracy of models using four different major gene markers and genome-wide association study (GWAS) markers as fixed effects. The prediction models used 31,975 markers that are replicated 50 times using a 5-fold cross-validation. We then compared GS models using a marker-assisted selection (MAS) to compare the prediction accuracy of the markers alone and in combination. GS models had higher accuracies than MAS and reached an accuracy of 0.72 for disease SEV. The major gene and GWAS markers had only a small to nil increase in the prediction accuracy more than the base GS model, with the highest accuracy increase of 0.03 for the major markers and 0.06 for the GWAS markers. There was a statistical increase in the accuracy using the disease SEV trait, BLs, population type, and combining years. There was also a statistical increase in the accuracy using the major markers in the validation sets as the mean accuracy decreased. The inclusion of fixed effects in low prediction scenarios increased the accuracy up to 0.06 for GS models using significant GWAS markers. Our results indicate that GS can accurately predict quantitative disease resistance in the presence of major and minor genes.

## Introduction

Plant breeding programs select and improve both qualitative and quantitative traits. Qualitative traits are controlled by a few large-effect genes that are readily detectable and follow a Mendelian inheritance (Chen, 2013). In contrast, quantitative traits are controlled by several small-effect genes that are difficult to distinguish and controlled by quantitative trait loci (QTL; Bernardo, 2008). The genetic control of a trait determines the types of selection that will be most effective for improvement. However, disease resistance can be either a qualitative or a quantitative trait, and, therefore, the most effective method of improvement varies (Poland and Rutkoski, 2016). Breeding for disease resistance is a major goal for most breeding programs due to the effect of the disease on yield and quality performance.

Breeding for qualitative disease resistance is controlled by one or two large-effect alleles, called resistance (R) genes and further referred to as major genes (Agrios, 2005). Qualitative disease resistance generally follows a race-specific resistance and quickly degrades due to the rapid evolution of new pathogen races (Chen, 2005). Major gene pyramiding can reduce the possibility of major genes by combining multiple major genes to provide a more durable resistance to multiple pathogen races into a single line. Pyramiding is implemented through a marker-assisted selection (MAS) and has been an effective method for various crops (Wang et al., 2001, 2017; Pietrusińska et al., 2011; Bai et al., 2012; Jiang et al., 2012; Liu et al., 2016b; Singh et al., 2017). Successful implementation of major genes relies on identifying the useful sources of the genes, finding the linked markers, confirming the effect in different genetic backgrounds, and finally, deploying said major genes (Bernardo, 2008). Major gene implementation is further complicated when it comes to selecting multiple major genes simultaneously for gene pyramiding. A large population is needed to screen and select the lines with more than one gene in early generations while still maintaining enough lines to select for other traits in later generations (Poland and Rutkoski, 2016). The difficulty can be further attributed to unfavorable linkage and multiple major gene sources (Bernardo, 2008).

Breeding for quantitative resistance conferred by minor-effect genes or a combination of minor and major genes tends to produce a more durable resistance in breeding lines (BLs) because it relies on multi-resistant alleles. Breeding for quantitative resistance requires multiple breeding cycles to improve resistance gradually (Poland and Rutkoski, 2016). The breeding method for quantitative resistance is similar to the methodology used for other complex traits such as grain yield (Rutkoski et al., 2014; Poland and Rutkoski, 2016; González-Camacho et al., 2018). Similar to qualitative resistance, selecting for quantitative resistance can be completed throughout the breeding process, but disease resistance is commonly completed in earlier generations to select for other traits further in the program. Therefore, selecting for quantitative resistance in earlier generations can be difficult due to the lack of replication and environments. However, selecting for resistance in later generations reduces genetic gain due to the selection for other traits (Poland and Rutkoski, 2016). Both methods, therefore, reduce the effectiveness of breeding quantitative resistance. One such trait that displays both qualitative and quantitative resistance is stripe rust, also called yellow rust (Yr), caused by *Puccinia striiformis* Westend. f. sp. *tritici* Erikss.

Stripe rust is one of the most devastating diseases of wheat (*Triticum aestivum* L.) and is highly destructive in the western USA (Chen, 2005; González-Camacho et al., 2018; Liu et al., 2019). Stripe rust can cause more than 90% yield losses in fields planted with susceptible cultivars (Liu et al., 2020). The use of resistance varieties and the applications of fungicide are the primary methods to control stripe rust (Chen and Line, 1995; Liu et al., 2020). Stripe rust resistance is categorized into qualitative all-stage resistance (ASR) and quantitative adult-plant resistance (APR).

All-stage resistance is conferred by race-specific genes that are inherited qualitatively with a life span of ~3.5 years per gene (Case et al., 2014; Chen and Kang, 2017). There are more than 300 identified QTL conferring resistance to stripe rust (Wang and Chen, 2017). The identification of a large number of major genes shows numerous resistance alleles available for breeding purposes in various varieties and populations. Previously, major genes *Yr5* and *Yr15* have been shown to be effective against all races of the stripe rust pathogen in the USA (Wang and Chen, 2017). However, virulence to *Yr5* has been demonstrated in a few countries not including the USA (Wellings et al., 2009; Zhang et al., 2020; Kharouf et al., 2021; Tekin et al., 2021). Virulence to *Yr15* has only been documented in Afghanistan (Gerechter-Amitai et al., 1989). The virulence to these genes demonstrates the need to not rely on any single major gene to provide resistance in a cultivar.

Adult-plant resistance is usually a non-race-specific quantitative resistance that is associated with durable resistance with some genes being effective for more than 60 years (Chen, 2013). APR is often affected by temperature and also can be referred to as high-temperature adult-plant (HTAP) resistance, which is often controlled by more than one gene mainly with additive effect (Chen and Line, 1995; Chen et al., 1995; Liu et al., 2019). HTAP resistance is influenced by the temperature and age of the plants. As the temperature increases, the plant becomes more resistant, and rust development slows down (Chen, 2005). However, to confirm HTAP, greenhouse studies with different temperature ranges need to be conducted (Chen, 2005). HTAP resistance and APR are conferred by different loci with varying effects and often display partial resistance, making them difficult to incorporate into new cultivars (Chen and Line, 1995; Liu et al., 2019). Consequently, APR or HTAP resistance must be improved over multiple selection cycles as mentioned previously (Rutkoski et al., 2014; Poland and Rutkoski, 2016; González-Camacho et al., 2018). APR is generally expressed in the later stages of wheat, whereas ASR is expressed throughout the lifecycle of the plant (Wang and Chen, 2017). Therefore, it is difficult to identify APR genes due to the masking of their effect by ASR genes. The masking of ASR genes and the quantitative nature of APR genes result in much of the APR resistance in a population being uncharacterized. It is recommended to combine both ASR and APR genes to take advantage of both types of resistance limitations (Wang and Chen, 2017). The lack of ASR durability coupled with the challenge in identifying and breeding APR creates a unique opportunity for genomic selection (GS). In addition, major ASR genes are known to interact with APR and including them in GS models as fixed effect have increased prediction accuracy (Bernardo, 2014; Rutkoski et al., 2014; Arruda et al., 2016).

In many crops, the difficulty in selecting for qualitative and quantitative disease resistance (similar to stripe rust) creates an opportunity for GS to integrate quantitative resistance by accounting for small-effect alleles in the presence of large-effect major genes without the development and analysis of mapping populations and techniques (Poland and Rutkoski, 2016). The goal of this study was to determine the most accurate GS method to select for disease resistance in the presence of both major and minor genes. Wheat stripe rust was used as an example as most plant breeders try to capture the additive effects of both ASR and APR simultaneously. The identified GS approaches will be a valuable tool for breeders to facilitate cultivar and parental selection for accumulating favorable alleles for disease resistance in the presence of major and minor resistance genes (Rutkoski et al., 2014; Michel et al., 2017).

## Materials and Methods

### Phenotypic Data

Two training populations were used to compare the inclusion of fixed-effect markers in populations with different frequencies of stripe rust genes. The first training population consists of F_3 : 5_ and double-haploid soft white winter wheat BLs developed by the Washington State University (WSU) winter wheat breeding program. The BL population was evaluated for stripe rust in the unreplicated single-plot trials in Pullman and Lind, Washington planted in 2016, 2017, 2018, and 2020 growing seasons (Table 1). Due to the unreplicated nature of the single-plot trails, each trial consisted of unique lines, which resulted in a total of 2,630 lines for all years and locations. The year 2019 was not included due to the lack of adequate disease SEV in our trials. The BL population was previously selected for stripe rust resistance in headrow plots the year previous to unreplicated trials. Susceptible BLs in headrow plots were culled and not included in the BL population, which represents a prior selected, closely related BL population with similar pedigree sources of stripe rust resistance. The second training population consists of diverse association mapping panel [diversity panel (DP)] trials evaluated in unreplicated trials in Central Ferry and Pullman, Washington from 2013 to 2016 with the same 475 lines represented in each trial (Table 1). The mapping panel consists of varieties and BLs from at least six soft white winter wheat breeding programs in the Pacific Northwest (PNW) and represents diverse backgrounds with the potential sources of stripe rust resistance.

**Table 1:** Training populations for stripe rust IT and SEV in Central Ferry, Lind, and Pullman, WA, USA from 2013 to 2020.

**Population^**a**^**	**Year**	**Trials**	**Locations**	**Lines^**b**^**	**IT 1^**c**^**	**SEV 1^**d**^**	**IT 2**	**SEV 2**	**IT 3**	**SEV 3**
DP	2013	2	2	475	X	X	X	X	X	X
DP	2014	2	2	474	X	X	X	X	X	X
DP	2015	2	2	474	X	X	X	X	X	X
DP	2016	1	1	474	X	X	X	X	X	X
DP	2013–2014	4	4	475	X	X	X	X	X	X
DP	2013–2015	6	6	475	X	X	X	X	X	X
DP	2013–2016	7	7	475	X	X	X	X	X	X
BL	2016	2	2	304	X	X	X			
BL	2017	4	2	728	X	X	X	X	X	X
BL	2018	3	2	1,239	X	X	X	X	X	X
BL	2020	1	1	373	X	X				
BL	2016–2017	6	4	1,029	X	X	X	X	X	X
BL	2016–2018	9	6	2,262	X	X	X	X	X	X
BL	2016–2020	10	7	2,630	X	X	X	X	X	X

a *DP, Diversity panel; BLs, Breeding lines*.

b *Lines, Unique lines in the training population*.

c *IT, Infection type*.

d *SEV, Disease severity*.

*X, Indicates measurement recorded*.

The measured disease traits were stripe rust IT and SEV. The recordings of these traits were dependent on natural infection and stripe rust incidence at the time of observation and were not previously inoculated. Some trials had three observations for stripe rust and were identified with sequential numbers. The first recording was taken soon after the emergence of a flag leaf, the second was taken again after anthesis, and the third was taken in the early milk stage. The trials with only one observation were recorded right after anthesis for responses in the adult plant stage as stripe rust was not present in the field during earlier growth stages. IT was recorded based on a 0–9 scale (Line and Qayoum, 1992). SEV was recorded as the percentage of the leaf-infected area using the modified Cobb Scale (Peterson et al., 1948). Table 1 summarizes environments, years, genotyped individuals, and the measurements taken for each trial during which stripe rust was recorded. However, due to the nature of APR being effective in the adult stage and the fact that not all trials had multiple recordings, only the last observation for each trial was used to measure the disease traits for APR.

To account for differences in disease pressure under different environments, a two-step adjusted mean method by which a linear model was implemented to adjust both IT and SEV means within and across environments was used. Then, a mixed linear model was used to calculate genomic estimated breeding values (GEBVs; Ward et al., 2019). Means from the stripe rust data collected in the unreplicated trials were adjusted using the residuals calculated for the unreplicated genotypes in individual environments and across environments using the modified augmented complete block design (ACBD) model (Federer, 1956; Goldman, 2019). The adjustments were made according to the method implemented in Merrick and Carter (2021), with the full model across environments as follows:

(1) $$\begin{array}{l} {\text{Y}}_{ij}={\text{Block}}_{i}+{\text{Check}}_{j}+{\text{Env}}_{k}+{\text{Block}}_{i}\;\times \;{\text{Env}}_{k}+{\text{Check}}_{j}\times \;{\text{Env}}_{k}+\varepsilon_{ijk} \end{array}$$

where *Y*_*ij*_ is the trait of interest, either IT or SEV; *Block*_*i*_ is the fixed effect of the *i*th block and *k*th trial; *Check*_*j*_ is the fixed effect of the *j*th replicated check cultivar; *Env*_*k*_ is the fixed effect of the *k*th trial; and ε_*ijk*_ denote the residual errors.

Heritability on a genotype-difference basis for broad-sense heritability was calculated using the variance components from the models implemented in Merrick and Carter (2021) and using the best linear unbiased predictors for both individual environments and across environments with the formula:

(2) $$\begin{array}{l} H_{Cullis}^{2}=1-\frac{{\bar{v}}_{\Delta ..}^{BLUP}}{2\sigma_{g}^{2}} \end{array}$$

where $\sigma_{g}^{2}$ and ${\bar{v}}_{\Delta }^{BLUP}$ are the genotype variance and mean variance of the BLUPs, respectively (Cullis et al., 2006). In general, the broad-sense heritability measurement is not suitable for an unreplicated, unbalanced multi-environment trial, and, therefore, narrow-sense heritability was not calculated (Schmidt et al., 2019).

### Genotypic Data

Lines were genotyped using genotyping-by-sequencing (GBS; Elshire et al., 2011) through the North Carolina State Genomics Sciences Laboratory in Raleigh, NC, USA, using the restriction enzymes *Msp*I and *Pst*I (Poland et al., 2012). Genomic DNA was isolated from seedlings in the one-leaf to three-leaf stage using Qiagen BioSprint 96 Plant kits and the Qiagen BioSprint 96 workstation (Qiagen, Germantown, MD, USA). DNA libraries were prepared following the protocol of DNA digestion with *Pst*I and *Msp*I restriction enzymes (Poland et al., 2012). Genotyping-by-sequencing (GBS; Elshire et al., 2011) was conducted at North Carolina State University Genomic Sciences Laboratory with either an Illumina HiSeq 2500 or a NovaSeq 6000. DNA library barcode adapters, DNA library analysis, and sequence single-nucleotide polymorphism (SNP) calling were provided by the USDA Eastern Regional Small Grains Genotyping Laboratory (Raleigh, NC, USA). Sequences were aligned to the Chinese Spring International Wheat Genome Sequencing Consortium (IWGSC) RefSeq v1.0 (Appels et al., 2018), using the Burrows–Wheeler Aligner (BWA) 0.7.17 (Li and Durbin, 2009). Genetic markers with more than 20% missing data, a minor allelic frequency of <5%, and the markers that were monomorphic were removed. Markers were then imputed using Beagle version 5.0 and filtered once more for markers less than a 5% minor allelic frequency (Browning et al., 2018). A total of 31,975 SNP markers for the 475 unique DP lines and 2,630 BLs were obtained from GBS. Principal components for the markers were calculated using the function “prcomp,” and a biplot with *k*-mean clusters was created using the function “autoplot” in R (R Core Team, 2018). Cluster number for *k*-means was calculated according to the elbow method using a screen plot with the identification of the optimal number of clusters when the total intracluster variation was minimized.

Major rust-resistant genes observed to be common in the WSU breeding population are *Yr10, Yr17, Lr68*, and *Qyr.wpg-1B.1*, and molecular marker data for these genes were included as fixed effects in our GS models. All winter wheat lines were genotyped using Kompetitive Allele Specific PCR (KASP®) assay for *Yr17, Lr68*, and *Qyr.wpg-1B.1* in the WSU winter wheat breeding laboratory. The *Yr17* gene (Helguera et al., 2003) was screened using the KASP marker developed by Milus et al. (2015). The *Lr68* leaf rust resistance gene (Herrera-Foessel et al., 2012) was screened using the KASP marker developed by Rasheed et al. (2016). Although leaf rust resistance is not commonly selected in the US PNW breeding programs, this gene was found in a large proportion of BLs, and thus was hypothesized that it might have been selected congruently with stripe rust resistance. The APR QTL *Qyr.wpg-1B.1* reported on chromosome 1B by Naruoka et al. (2015) was screened using the marker *IWB12603* (Mu et al., 2020). The KASP assays were performed using PACE^TM^ Genotyping Master Mix (3CR Bioscience, Essex, UK) following the instructions of the manufacturer, and endpoint genotyping was conducted on fluorescence using a Lightcycler 480 Instrument II (Roche, Indianpolis, IN, USA). The previously reported ASR gene *Yr10* (Frick et al., 1998) was screened with a microsatellite marker *Xpsp3000* developed by Bariana et al. (2002). The microsatellite marker *Xpsp3000* was run using PCR products, which were separated on an ABI3730XL DNA fragment analyzer (Applied Biosystems, Waltham, MA, USA), and alleles were scored with the GeneMarkerv4.0 software (SoftGenetics, State College, PA, USA), in collaboration with the USDA Western Regional Small Grains Genotyping Laboratory in Pullman, Washington.

### Genome-Wide Association Model

In addition to the inclusion of molecular markers for major rust-resistant genes as fixed effects, the markers identified through genome-wide association studies (GWASs) were included through *de novo* GWAS. This method is further referred to as GWAS-assisted GS (GWAS-GS). The GWAS-GS was implemented according to McGowan et al. (2020). Briefly, a proper cross-validation using GWAS was conducted using BLINK in the genome association and prediction integrated tool (Liu et al., 2016a; Tang et al., 2016; Huang et al., 2019; GAPIT) with three principal components fitted as fixed effects on the training population only. Three principal components were used because they were previously observed to be most reliable in accounting for a population structure for yield and agronomic traits in winter wheat for the same populations (Lozada et al., 2017). In accordance to advice put forward by Rice and Lipka (2019), the first method of GWAS-GS included only significant markers based on a Bonferonni cutoff of 0.05 (GWAS_B). Due to our cross-validation scheme, different significant markers for GWAS_B were identified in each cross-validation, year, and population. Therefore, significant markers were not presented. For the remaining GWAS-GS methods, the markers were ordered by the degree of statistical significance based on the values of *p* from the smallest to largest. We compared the inclusion of the top 5, 10, 25, 50, and 100 most significant markers as fixed effects (GWAS_5, GWAS_10, GWAS_25, GWAS_50, and GWAS_100).

### Prediction Models

#### Marker-Assisted Selection Model

Single and multiple regression models were used as MAS models to compare major rust-resistant markers and the predictive ability of *de novo* GWAS markers alone and in combination. The fixed-effect multiple regression model is described as follows:

(3) $$\begin{array}{l} y_{i}=\mu +X_{1}\beta_{1}+\ldots X_{i}\beta_{i}+\varepsilon_{i} \end{array}$$

where *y*_*i*_ is the observed phenotypic value of the *i*th individual, μ is the mean, *X*_*i*_ is the genotype of the marker *i*, β_*i*_ is the effect of the *i*th marker, and ε_*i*_ is the residual error term.

#### GS Model

rrBLUP was used as the base GS model and was implemented using the package “rrBLUP” (Endelman, 2011). rrBLUP was used as the base model due to the nonplacement of the ridge regression penalty implemented by rrBLUP on the fixed effects, allowing a large effect on the model. Further, rrBLUP has shown to outperform other models when integrating fixed effects into the models and in predicting disease resistance (Rutkoski et al., 2014; Arruda et al., 2016; Poland and Rutkoski, 2016; Muleta et al., 2017). The basic rrBLUP model is described as follows (Rice and Lipka, 2019):

(4) $$\begin{array}{l} y_{i}=\mu +\overset{p}{\underset{k=1}{\sum }}x_{ik}\beta_{k}+\varepsilon_{i} \end{array}$$

where *y*_*i*_ is the observed phenotypic value of the *i*th individual, μ is the mean, *x*_*ik*_ is the genotype of the *k*th marker and *i*th individual, *p* is the total number of markers, β_*k*_ is the estimated random marker effect of the *k*th marker, and ε_*i*_ is the residual error term.

#### GS Model With Fixed Effects

To evaluate the effect of major and *de novo* GWAS markers on the prediction accuracy of GS models, we used the rrBLUP model as described (Rice and Lipka, 2019):

(5) $$\begin{array}{l} y_{i}=\mu +\overset{m}{\underset{j=1}{\sum }}x_{ij}\alpha_{j}\overset{p}{\underset{k}{\sum }}x_{ik}\beta_{k}+\varepsilon_{i} \end{array}$$

where *y*_*i*_ is the observed phenotypic value of the *i*th individual, μ is the mean, *x*_*ij*_ is the *j*th marker of the *i*th individual, *m* is the number of markers included as fixed-effect covariates, α_*j*_ is the fixed additive effect of the *j*th marker, *x*_*ik*_ is the genotype of the *k*th marker and *i*th individual, *p* is the total number of markers, β_*k*_ is the estimated random marker effect of the *k*th marker, and ε_*i*_ is the residual error term.

### Prediction Accuracy and Schemes

The prediction accuracy for the GS was reported using Pearson correlation coefficients, and a prediction bias was reported using a root mean square error (RMSE) between GEBVs and their respective adjusted means using the function “cor” in R (R Core Team, 2018). The effect of fixed-effect markers on prediction accuracy was assessed using a 5-fold cross-validation scheme and independent validation sets for IT and SEV in the DP and BL training populations. The two populations were used to compare the effects of the significant markers in populations with different genetic relatedness, frequency of markers, and sources of resistant pedigrees. GS models were conducted with 5-fold cross-validation by including 80% of the samples in the training population and predicting the GEBVs of the remaining 20% (Lozada and Carter, 2019). One replicate consists of the five model iterations, where the population is split into five different groups. This was completed 50 times. As mentioned previously for the GWAS-GS, the GWAS was conducted on 80% of the lines, and then the markers are included in the GS model to predict the remaining 20% of the lines. Independent validation sets were then performed on a yearly basis by combining the two training populations and environments together per year. This allows the evaluation of models in a realistic breeding situation in which we combine all available data to build a training population.

The training populations were evaluated for cross-validations on a yearly basis and over combined years and trials. We assessed each year independently using cross-validations. We then created prediction models starting with the earliest trial and then a new model with the addition of each subsequent trial to evaluate a genotype-by-environment interaction, continuous training of a prediction model, and the effect of different races of *P. striiformis* f. sp. *tritici*. Independent validation sets were first conducted using continuous training. For example, the earliest year, i.e., 2013, was used to predict the following year, i.e., 2014. The years were then combined to predict the following year, i.e., 2013 and 2014 to predict 2015, and this process was continued until the years 2013–2018 were used to predict 2020. Using this scenario, the first 3 years, 2013–2015, consisted of the DP lines alone, and therefore, each year consisted of the same lines. With the inclusion of the years 2016–2020, unique lines from the BL were added each year due to the fact that each trial in the BL consisted of unique lines only phenotyped in a single trial as mentioned previously.

All GS and MAS models and scenarios were analyzed using WSU's Kamiak high performance computing cluster (Kamiak, 2021). Model and year comparisons were evaluated by using a Tukey's honestly significant difference (HSD) test implemented in the “agricolae” package in R (R Core Team, 2018; de Mendiburu and de Mendiburu, 2019). The comparison of models was then plotted for a visual comparison using “ggplot2” in R (Wickham, 2011; R Core Team, 2018).

## Results

### Phenotypic Data

Stripe rust phenotyping was dependent on natural infection. Therefore, it is important to evaluate GS models in different years to account for environments with little to no variation in stripe rust SEV and pathogen race changes. Overall, the maximum IT and SEV were relatively high for each scale, indicating the presence of adequate stripe rust SEV in each trial (Table 2). The BL had relatively high coefficient of variations (CVs) for each trial. However, the heritability was very high, ranging from 0.60 to 0.96 across traits and trials, indicating adequate screening trials for stripe rust. Further, the phenotypic correlations between IT and SEV were relatively high in the DP, ranging from 0.67 in 2013 to 0.88 in 2015 (Table 3). The phenotypic correlation in the BL between IT and SEV was similarly high, ranging from 0.70 in 2016 to 0.86 in 2018.

**Table 2:** Stripe rust IT and disease SEV heritability (H^2^) and trial statistics for unadjusted phenotypes in the DP and BL training population phenotypes from 2013 to 2016 and 2016 to 2020.

**Population**	**Year**	**Trait**	**H^**2**^**	**CV^**a**^**	**Max^**b**^**	**Mean**	**Min^**c**^**	**SD^**d**^**
DP	2013	IT	0.85	52.31	9	3	1	2
DP	2014	IT	0.82	57.13	9	4	1	2
DP	2015	IT	0.89	43.82	9	5	1	2
DP	2016	IT	0.84	46.23	9	4	0	2
DP	2013–2014	IT	0.93	55.58	9	4	1	2
DP	2013–2015	IT	0.94	52.66	9	4	1	2
DP	2013–2016	IT	0.95	51.77	9	4	0	2
DP	2013	SEV	0.91	108.31	100	22	2	24
DP	2014	SEV	0.78	116.04	90	24	2	28
DP	2015	SEV	0.92	72.78	100	43	2	32
DP	2016	SEV	0.89	70.57	100	36	0	26
DP	2013–2014	SEV	0.93	112.77	100	23	2	26
DP	2013–2015	SEV	0.96	99.31	100	30	2	30
DP	2013–2016	SEV	0.96	94.90	100	31	0	29
BL	2016	IT	0.90	87.56	8	3	0	2
BL	2017	IT	0.83	83.36	9	3	0	2
BL	2018	IT	0.79	172.49	8	2	0	3
BL	2020	IT	0.96	93.03	8	3	0	3
BL	2016–2017	IT	0.83	84.06	9	3	0	2
BL	2016–2018	IT	0.79	115.26	9	2	0	3
BL	2016–2020	IT	0.80	113.22	9	2	0	3
BL	2016	SEV	0.86	152.31	80	9	0	13
BL	2017	SEV	0.88	131.79	90	16	0	21
BL	2018	SEV	0.60	212.74	80	11	0	24
BL	2020	SEV	0.96	125.06	80	18	0	23
BL	2016–2017	SEV	0.89	136.36	90	15	0	20
BL	2016–2018	SEV	0.85	165.25	90	13	0	22
BL	2016–2020	SEV	0.86	160.47	90	14	0	22

a *CV, Coefficient of variation*.

b *Max, Maximum*.

c *Min, Minimum*.

d *SD, Standard deviation*.

**Table 3:** Phenotypic correlations between IT and disease SEV.

**Population**	**Year 1**	**Year 2**	**Year 3**	**Year 4**	**Year 1–2**	**Year 1–3**	**Year 1–4**
DP	0.79	0.67	0.88	0.82	0.76	0.85	0.86
BL	0.70	0.80	0.86	0.85	0.76	0.83	0.83

*Phenotypic correlations between IT and disease SEV for Pacific Northwest (PNW) winter wheat within both the diversity panel (DP) lines and breeding lines (BL) phenotyped from 2013 to 2020 in Central Ferry, Lind, and Pullman, WA, USA*.

**Figure 1:**
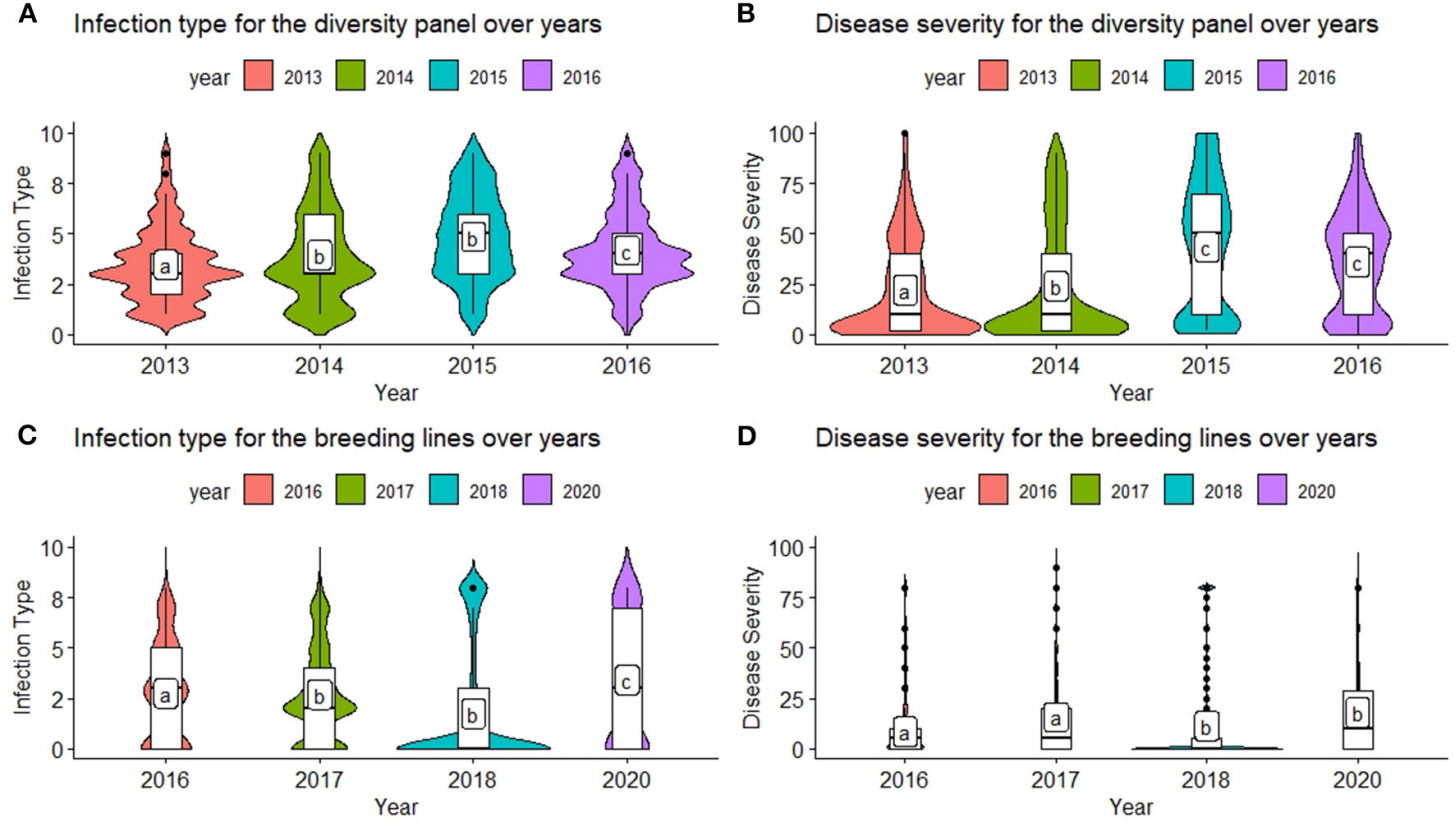
Comparison of infection type (IT) and disease severity (SEV) over years in the diversity panel (DP) lines and breeding lines (BL) training populations using least significant differences. Models labeled with the same letter are not significantly different (*p* = 0.05). **(A)** Infection type for the diversity panel over years. **(B)** Disease severity for the diversity panel over years. **(C)** Infection type for the breeding lines over years. **(D)** Disease severity for the breeding lines over years.

The inclusion of multiple environments creates a challenge for GS models due to the genotype–environment interaction (GEI). There were significant differences between the majority of years for each population and trait (Figures 1A–D). The ranges for both IT and SEV were large, indicating both resistant and nonresistant varieties within the populations. The mean IT and SEV were also lower in the BL compared to the DP (Figures 1A–D; Table 2). In comparison to the DP, the BL population consisted of a larger proportion of resistant cultivars, which was expected as these had previously been selected under field conditions. SEV displayed a large concentration of values near zero, specifically in the year 2018 (Figure 1D). Significant differences of each year indicate an environmental effect that needs to be accounted for within the prediction models.

In addition to GEI, stripe rust races may change from year to year, which creates an opportunity for major genes to be overcome by virulent races. The USDA stripe rust lab records race frequencies and virulence each year (https://striperust.wsu.edu/races/data/). The major stripe rust races for each year was either PSTv-37 or PSTv-52 with the exception of 2017 and 2020, which had large frequencies for PSTv-37 (Supplementary Table 1). The other races with higher frequencies included PSTv-39, PSTv-322, PSTv-48, PSTv-79, PSTv-11, and PSTv-73. Therefore, the difference in race change was not a major factor in prediction scenarios.

### Genotypic Data

**Figure 2:**
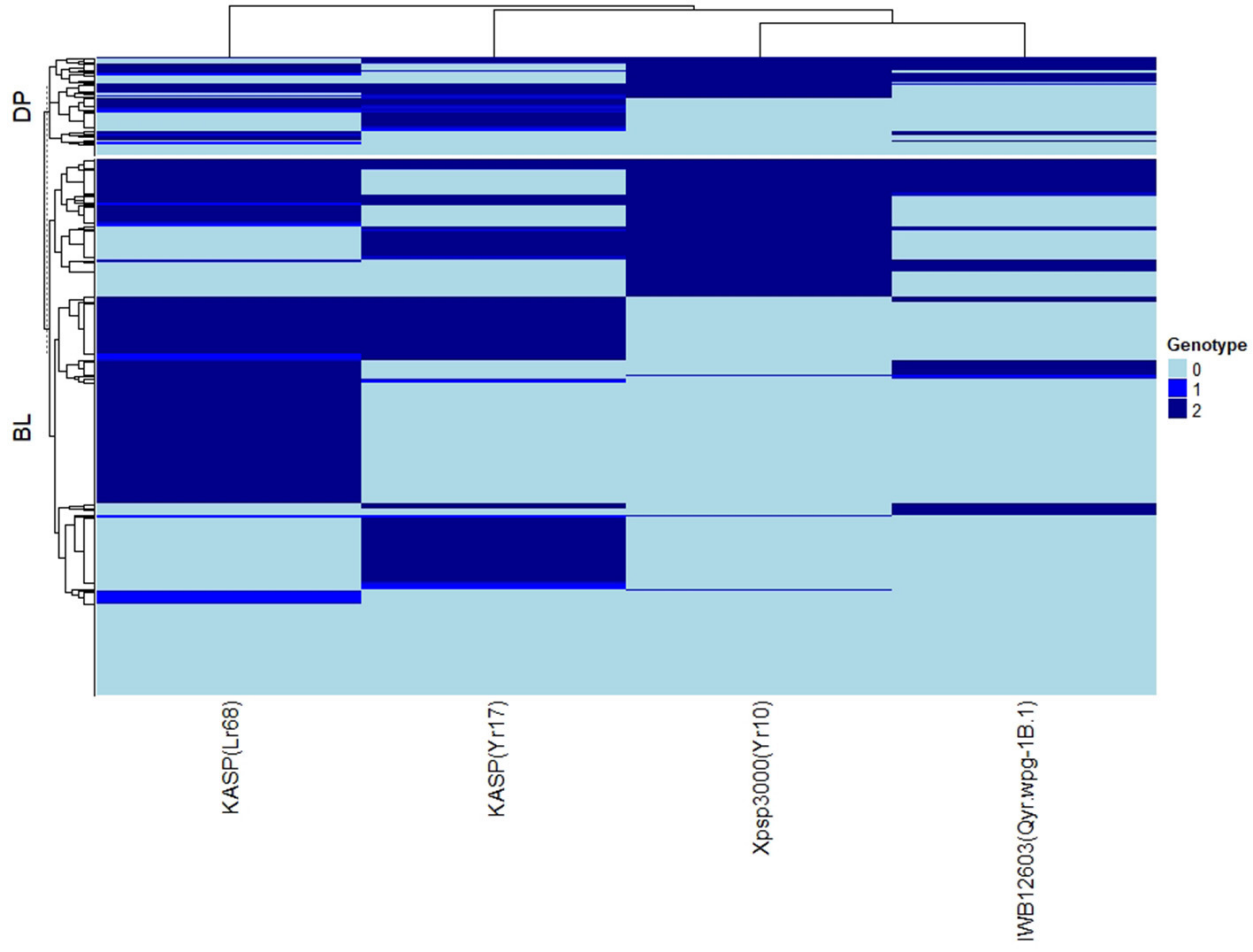
Heat map and hierarchical clustering for lines in the diversity panel (DP) lines and breeding lines (BL) populations for the major rust markers: IWB12603(Qyr.wpg-1B.1), KASP(Lr68), Xpsp3000(Yr10), and KASP(Yr17). Genotype: 0, homozygous wild-type allele; 1, heterozygous with both alleles present; 2, homozygous resistant allele.

The major rust genes present in the WSU winter wheat breeding program germplasm are *Yr10, Yr17, Lr68*, and *Qyr.wpg-1B*. The frequency of genotypes, as determined by the previously described molecular markers for each of these genes, is presented in Table 4. Similar frequencies in both populations were observed for the homozygous resistant allele for *Lr68* with 50 and 46% in the DP and BL, respectively. The frequency of the marker for *Yr10* and *IWB12603* was much higher in the DP than in the BL with *Yr10* having a relatively high frequency of 53% in the DP. However, the homozygous resistant allele for *Yr17* was much higher in the BL (38%) than in the DP (19%). There was also a wide combination of homozygous resistant alleles within each population (Figure 2).

**Table 4:** Frequency of rust-resistant genotypes^a^ in both the breeding line (BL) and diversity panel (DP) line populations.

**Population**	**Marker (gene)**	**Genotype**	**Numbe^**b**^**	**Frequency**	**Major race effectiveness**
DP	KASP (*Yr17*)	0	356	0.75	PSTv-322 PSTv-48 PSTv-79 PSTv-11
		1	30	0.06	
		2	89	0.19	
DP	IWB12603 (Qyr.wpg-1B.1)	0	288	0.61	NA^c^
		1	16	0.03	
		2	171	0.36	
DP	KASP (*Lr68*)	0	182	0.38	NA
		1	55	0.12	
		2	238	0.50	
DP	Xpsp3000 (*Yr10*)	0	220	0.46	PSTv-37 PSTv-52 PSTv-322 PSTv-48 PSTv-79 PSTv-11 PSTv-73
		1	4	0.01	
		2	251	0.53	
BL	KASP (*Yr17*)	0	1,491	0.57	PSTv-322 PSTv-48 PSTv-79 PSTv-11
		1	131	0.05	
		2	1,008	0.38	
BL	IWB12603 (Qyr.wpg-1B.1)	0	2,244	0.85	NA
		1	53	0.02	
		2	333	0.13	
BL	KASP (*Lr68*)	0	1,255	0.48	NA
		1	166	0.06	
		2	1,209	0.46	
BL	Xpsp3000 (*Yr10*)	0	2,172	0.83	PSTv-37 PSTv-52 PSTv-322 PSTv-48 PSTv-79 PSTv-11 PSTv-73
		1	11	0.00	
		2	447	0.17	

a *Genotype: Allele 0: homozygous wild-type allele; Allele 1: heterozygous with both alleles present; Allele 2: homozygous resistant allele*.

b *Number, number of lines*.

c *NA, Data were not available*.

**Figure 3:**
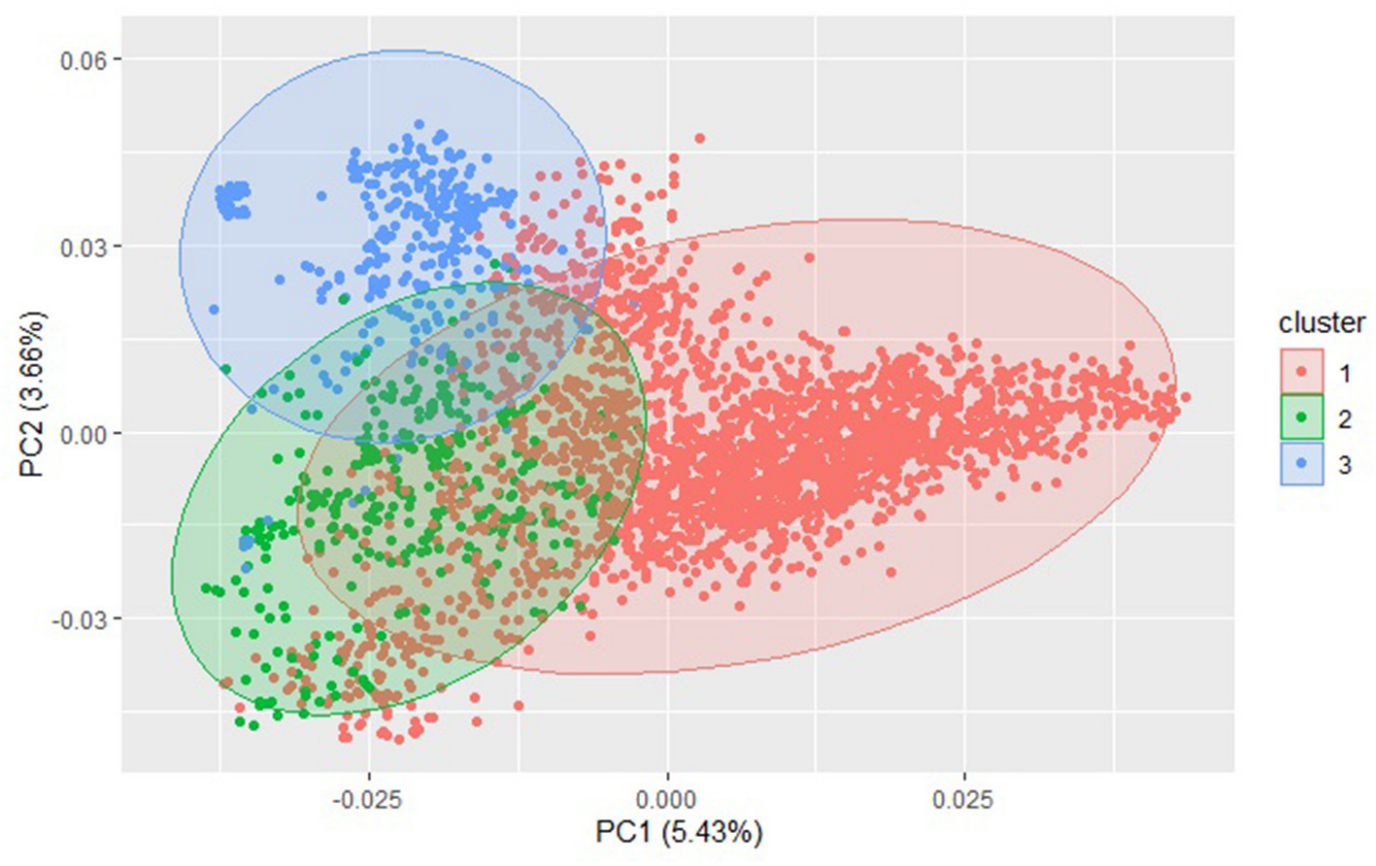
Principal component (PC) biplot and *k*-means clustering of single-nucleotide polymorphism (SNP) genotyped-by-sequencing (GBS) markers from the diversity panel (DP) lines and breeding lines (BL) training populations.

The principal component biplot using the GBS SNP markers over the combined DP and BL training populations accounted for only 9.1% of the total genetic variation, indicating a large population structure (Figure 3). PC1 explained 5.4% of the variation, and PC2 explained 3.7% of the variation. The biplot revealed three main clusters over the combined populations using *k*-means clustering. A majority of lines in both the DP and BL were included in the first cluster with 355 and 2,107 lines, respectively. The second cluster also displayed a mixture of DP lines and BLs with 108 and 219 lines, respectively. Finally, the third cluster included mainly BLs with 12 lines in the DP and 304 lines from the BL.

### Cross-Validations

#### Major Markers

**Figure 4:**
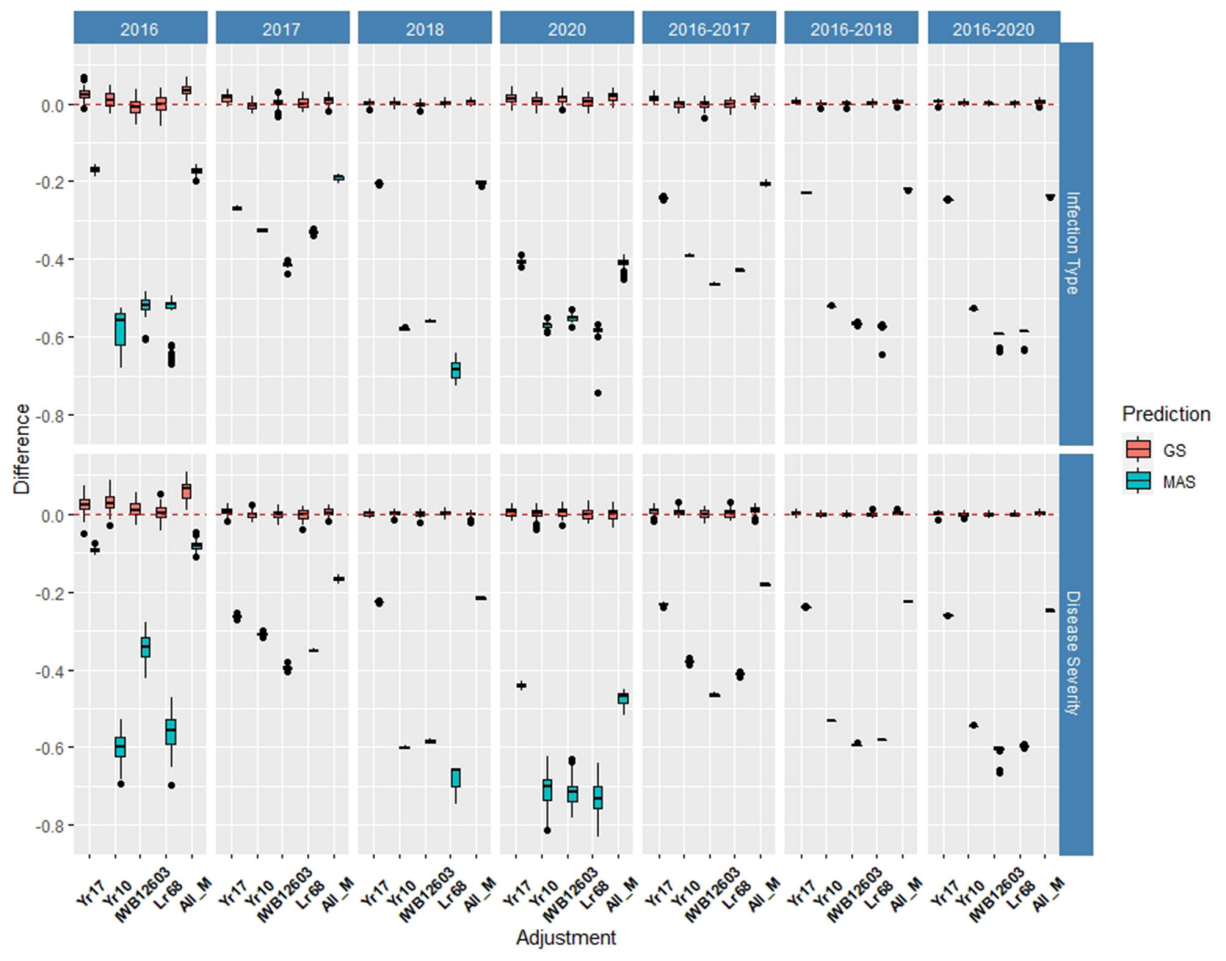
Difference in prediction accuracy from the base rrBLUP model for major markers in genomic selection (GS) and marker-assisted selection (MAS) using cross-validations in the BLs phenotyped from 2016 to 2020. Adjustments: ALL_M, IWB12603(Qyr.wpg-1B.1), KASP(Lr68), Xpsp3000(Yr10), and KASP(Yr17) combined.

**Figure 5:**
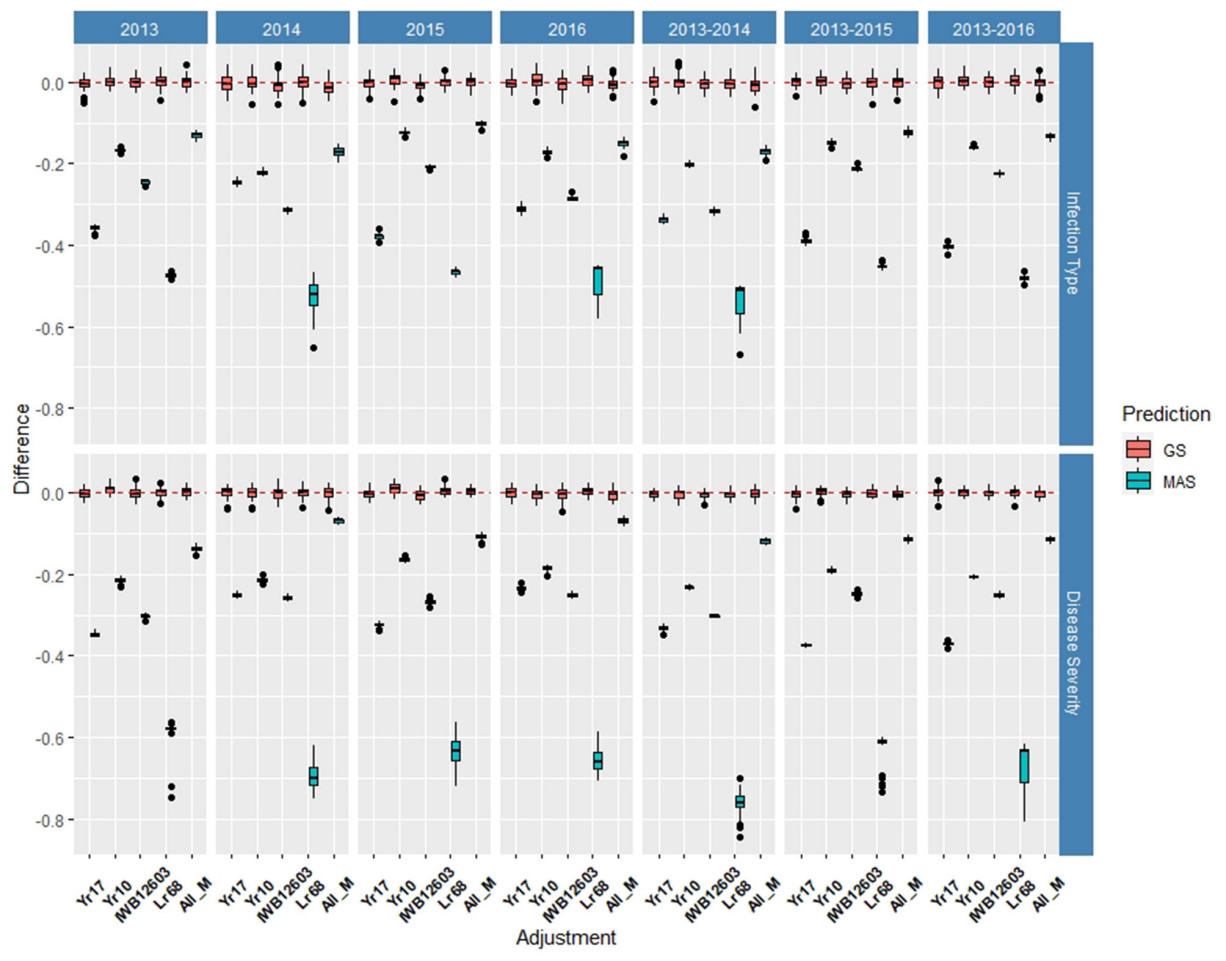
Difference in prediction accuracy from the base rrBLUP model for major markers in GS and MAS using cross-validations in the diversity panel (DP) lines phenotyped from 2013 to 2016. Adjustments: ALL_M, IWB12603(Qyr.wpg-1B.1), KASP(Lr68), Xpsp3000(Yr10), and KASP(Yr17) combined.

Multiple comparisons for accuracy and RMSE between the inclusion of each major molecular marker for the known rust-resistant genes individually and in combination (ALL_M) were completed for both populations in and across the years for IT and SEV (Supplementary Tables 2, 3). The markers for major rust genes were included as fixed effects and compared to the base rrBLUP model and MAS models with the markers as variables alone (Figures 4, 5; Supplementary Tables 4, 5). Within individual years in the BL, the rrBLUP base model reached a high accuracy of 0.65 in 2018 and 2020 for IT and 0.68 in 2018 for SEV. The effects of the major markers varied from year to year, but the marker for *Yr17* showed an increase in the prediction accuracy for every year except in 2018 for IT (Supplementary Table 2) and in 2018 and across 2016–2018 and 2016–2020 for SEV (Supplementary Table 3). A majority of markers had relatively low prediction accuracies for MAS with the exception of the *Yr17* marker that reached an accuracy of 0.05 and 0.42 for IT and SEV, respectively (Supplementary Tables 4, 5). When all markers were combined, similar accuracies were attained and compared to the time of inclusion of only the *Yr17* marker in both the rrBLUP model and MAS models. The remainder of major rust gene markers with the exception of *Lr68* increased the accuracy within specific years but had less consistency than the *Yr17* marker for. The largest differences from the rrBLUP model within a single year in the BL were seen in 2016 for GS models (Supplementary Figure 1). In 2016, the combination of both *Yr10* and *Yr17* markers increased the accuracy by 0.06 for SEV. *Yr17* and the combination of markers only slightly increased the accuracy across environments with an increase in IT of 0.01 (Supplementary Figure 2). Additionally, the RMSE was similar between all markers and rrBLUP for both traits (Supplementary Figure 3; Supplementary Tables 2, 3). However, for MAS, RMSE was higher than all GS models. In MAS models, the RMSE was lower for *Yr17*, and ALL_M, compared to another marker, with SEV having a much higher error than IT for the majority of years. The individual years of 2018 and 2020 displayed a higher RMSE compared to the other individual years and combined years (Supplementary Figure 3; Supplementary Tables 4, 5).

Within individual years in the DP, the rrBLUP base model reached an accuracy of 0.55 for IT (Supplementary Table 2) and 0.64 for SEV in 2013 (Supplementary Table 3). Across years, IT reached 0.56 in 2013–2016 (Supplementary Table 2) and SEV reached 0.69 in 2013–2014 (Supplementary Table 3). In the DP, the major rust markers had a less effect on the prediction accuracy, with *Yr10* being the only marker that increased the accuracy from the base rrBLUP model and at a maximum of 0.01. For MAS, the combination of markers resulted in the least reduction of accuracy with a maximum reduction of 0.10 in 2015 for IT (Supplementary Table 4) and 0.07 in 2016 for SEV (Supplementary Table 5). Markers for *Yr10* and IWB12603 also had the largest effect on MAS models. The largest differences from the rrBLUP model within a single year in the DP were seen in 2015 for GS models (Supplementary Figure 4). In 2015, the *Yr10* marker increased the accuracy by 0.01. There were no increases in the accuracy across any combination of environments in the DP. The results for RMSE were similar to the BL, with SEV having a much higher RMSE for each model than IT (Supplementary Tables 2, 3). Further, within SEV for MAS, *Lr68* had a higher RMSE compared to the other markers. *Yr17* did not display a lower RMSE than the other markers, with *Yr10* and ALL_M displaying the lowest RMSE in MAS (Supplementary Figure 5; Supplementary Tables 4, 5).

#### *De novo* GWAS Markers

**Figure 6:**
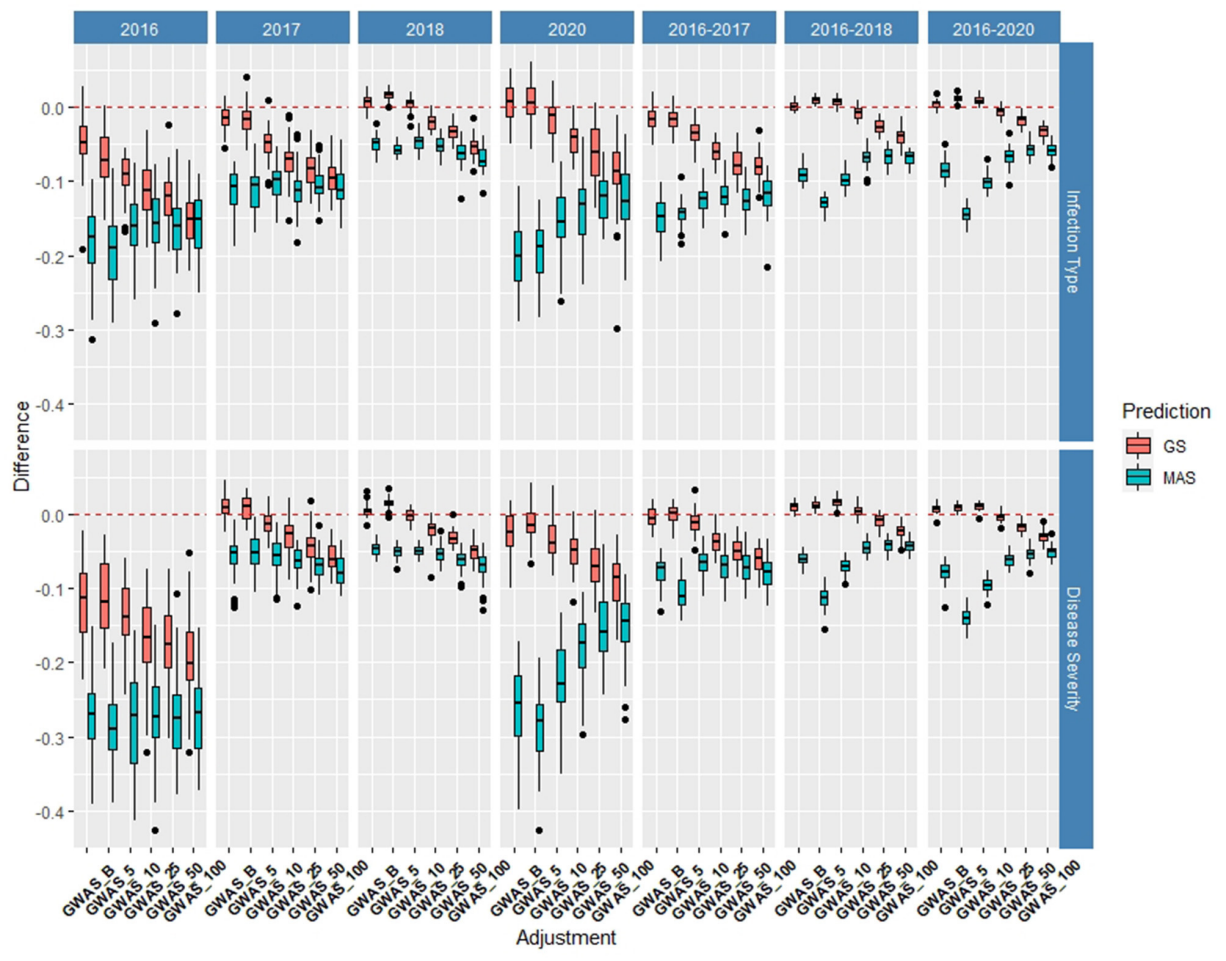
Difference in prediction accuracy from the base rrBLUP model for *de novo* genome-wide association study (GWAS) markers in GS and MAS using cross-validations in the BLs phenotyped from 2016 to 2020. Adjustments: GWAS_B, genome-wide association study assisted GS (GWAS-GS) with Bonferonni significant markers; GWAS_5, GWAS-GS with the top five significant markers; GWAS_10, GWAS-GS with the top 10 significant markers; GWAS_25, GWAS-GS with the top 25 significant markers; GWAS_50, GWAS-GS with the top 50 significant markers; GWAS_100, GWAS-GS with the top 100 significant markers.

**Figure 7:**
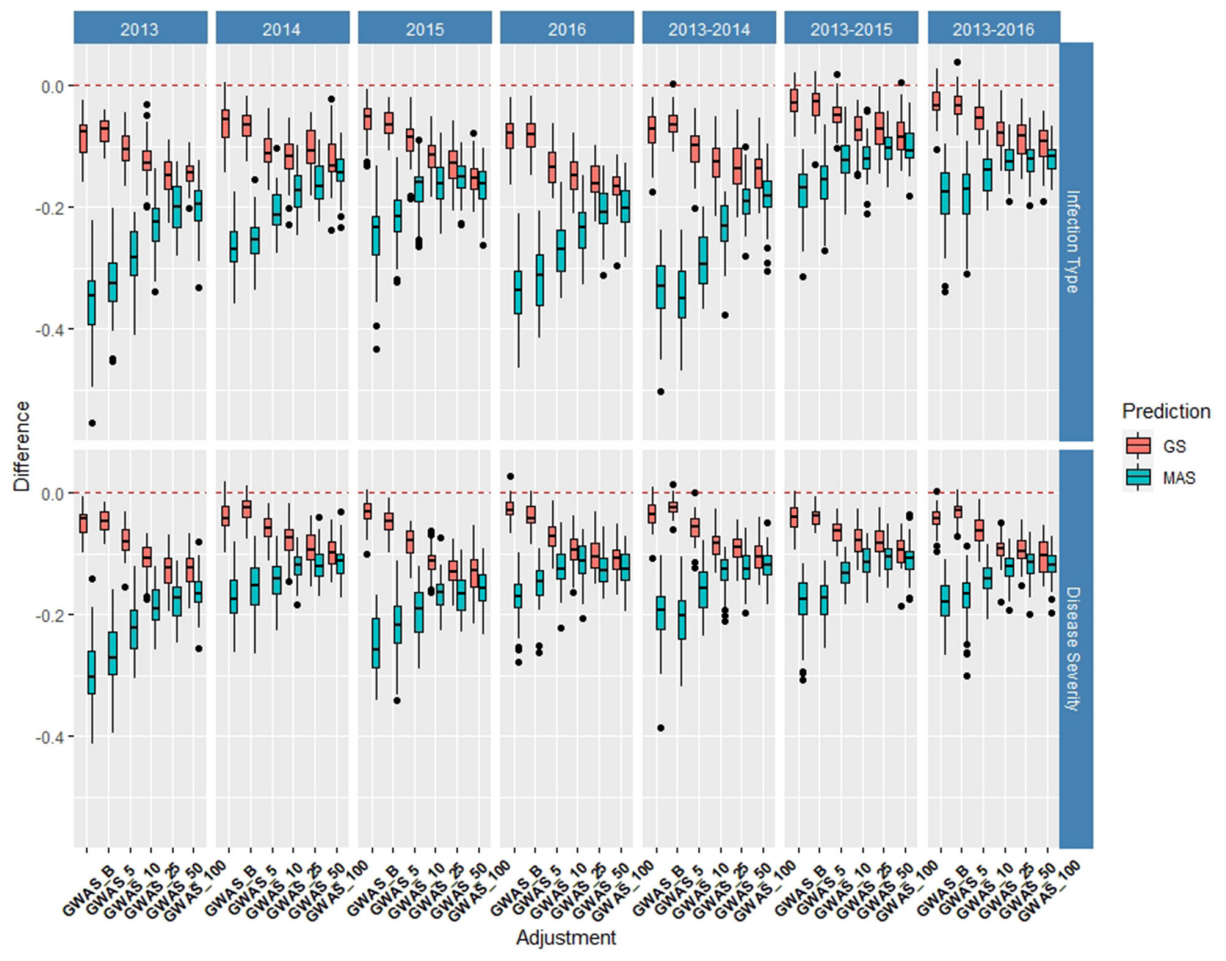
Difference in prediction accuracy from the base rrBLUP model for *de novo* GWAS markers in GS and MAS using cross-validations in the diversity panel (DP) lines phenotyped from 2013 to 2016. Adjustments: GWAS_B, GWAS-GS with Bonferonni significant markers; GWAS_5, GWAS-GS with the top five significant markers; GWAS_10, GWAS-GS with the top 10 significant markers; GWAS_25, GWAS-GS with the top 25 significant markers; GWAS_50, GWAS-GS with the top 50 significant markers; GWAS_100, GWAS-GS with the top 100 significant markers.

The *de novo* GWAS markers increased the prediction accuracy in individual years and across years in the BL, but not in the DP. Only the GWAS_B, GWAS_5, and GWAS_10 sets increased the accuracy with GWAS_25, GWAS_50, and GWAS_100 decreasing the prediction accuracy (Figures 6, 7). The largest increase in IT was for GWAS_5 in 2018 with an increase of 0.02 for both IT and SEV (Supplementary Tables 1, 2; Supplementary Figure 6). Across years, GWAS_10 had the largest increase of 0.02 in 2016–2018 for SEV (Supplementary Table 3; Supplementary Figure 7). The MAS for the *de novo* GWAS markers had larger decreases in MAS compared to the major markers in both the DP and BL (Supplementary Tables 4, 5). The larger GWAS sets (GWAS_25, GWAS_50, and GWAS_100) consistently had lower prediction accuracies than the other GWAS sets and the major rust gene markers. GWAS_B using significant markers showed the similar accuracies to GWAS_5, displaying no advantage compared to arbitrarily including markers based on the value of *p*. The GWAS-GS models displayed a higher RMSE for both GS and MAS in both population and traits compared to the major markers (Supplementary Figures 8, 9). The GWAS_100 sets displayed the highest RMSE out of all models in the cross-validation scenarios with an RMSE of 43.02 (Supplementary Table 5).

### Validation Sets

#### Major Markers

**Figure 8:**
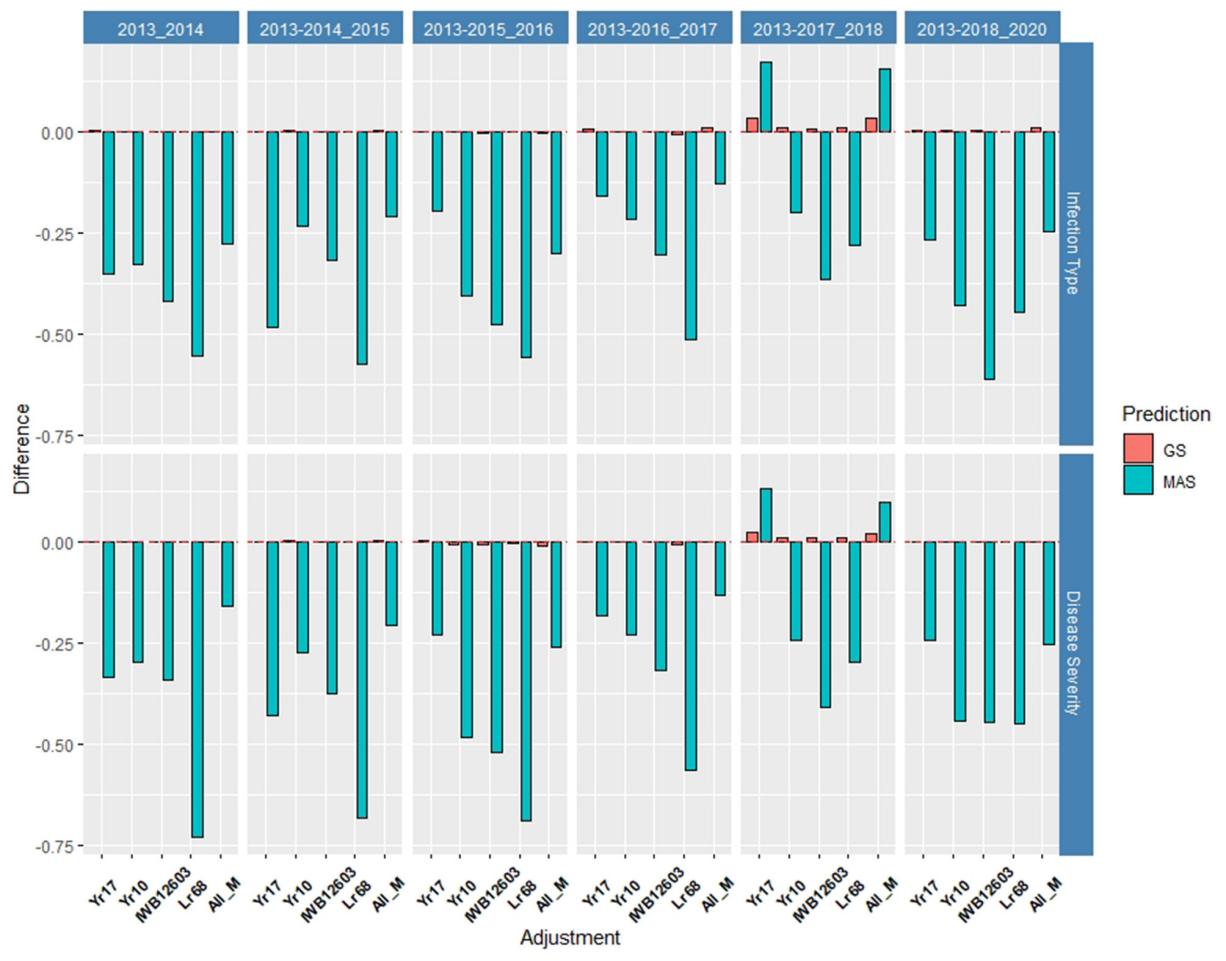
Difference in prediction accuracy from the base rrBLUP model for major markers in GS and MAS in the validation set using the diversity panel (DP) lines and breeding lines (BL) phenotyped from 2013 to predict 2020. Adjustments: ALL_M, IWB12603(Qyr.wpg-1B.1), KASP(Lr68), Xpsp3000(Yr10), and KASP(Yr17) combined.

**Figure 9:**
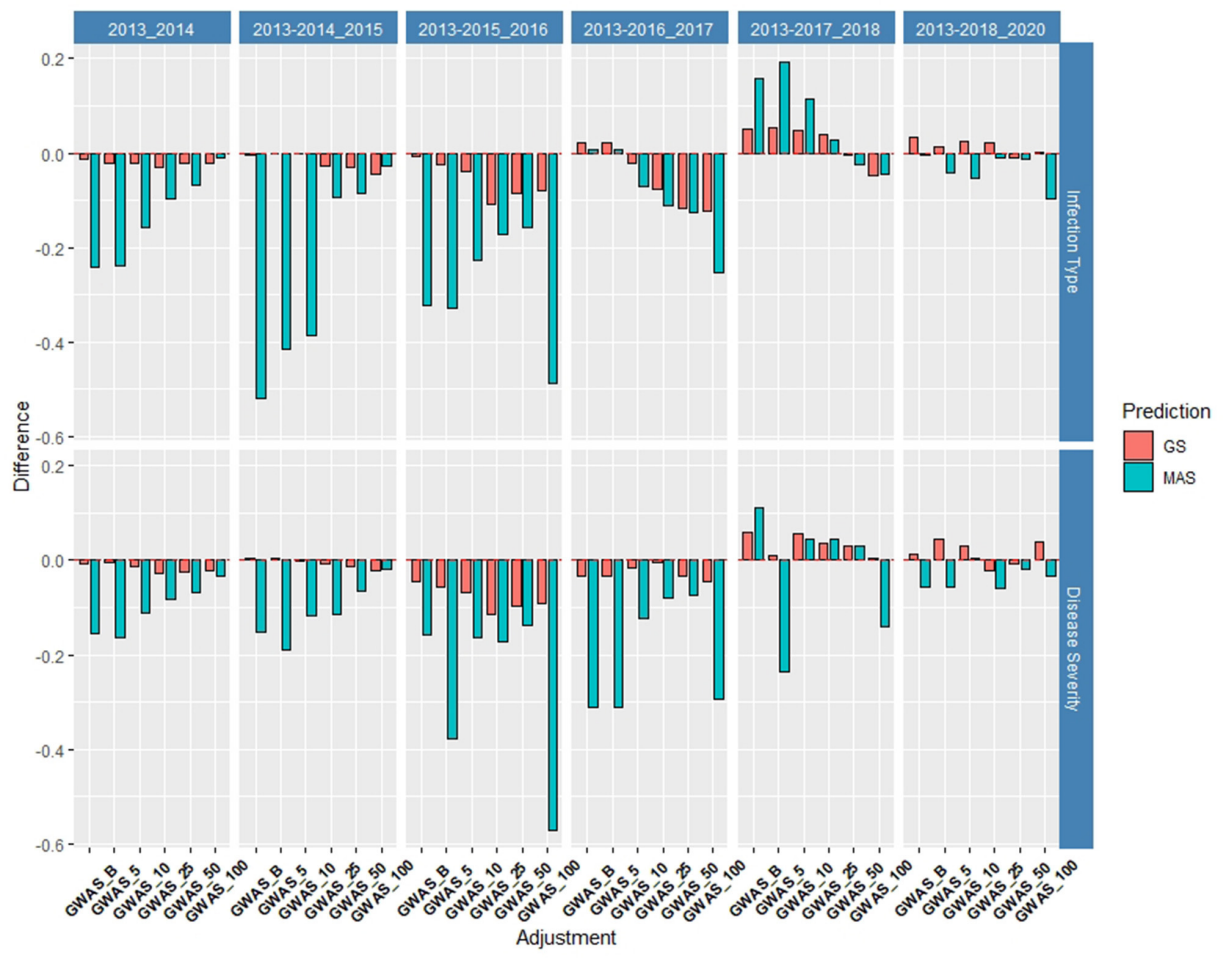
Difference in prediction accuracy from the base rrBLUP model for *de novo* GWAS markers in GS and MAS in the validation set using the diversity panel (DP) lines and breeding lines (BL) phenotyped from 2013 to predict 2020. Adjustments: GWAS_B, GWAS-GS with Bonferonni significant markers; GWAS_5, GWAS-GS with the top five significant markers; GWAS_10, GWAS-GS with the top 10 significant markers; GWAS_25, GWAS-GS with the top 25 significant markers; GWAS_50, GWAS-GS with the top 50 significant markers; GWAS_100, GWAS-GS with the top 100 significant markers.

The validation sets were conducted by combining both training populations and years and predicting the following year as a forward prediction. In doing so, the validation sets were evaluated to demonstrate real-world breeding scenarios wherein all available information was used to create predictions. The first 3 years, 2013–2015, consisted exclusively of the DP, and from 2016 forward, the BL was included due to the availability of training populations. The validation sets resulted in the highest accuracy of all prediction scenarios using the rrBLUP base model, and all major markers reached an accuracy of 0.72 in the SEV for predicting 2014 using the 2013 data (Figures 8, 9; Supplementary Tables 6, 7). For the same year, the major markers with the exception of *Yr10* resulted in an increase of the accuracy by 0.01. The major rust markers either performed the same or increased the accuracy for the majority of validation GS predictions.

As a number of environments and years were added to the population, the general prediction accuracy decreased presumably due to the prediction of multiple environments within a year and the inclusion of different training populations. However, as the accuracy decreased for the base rrBLUP model, the effect of fixed markers increased. The largest increase in both cross-validations and validation sets occurred using 2013–2017 to predict 2018, resulting in MAS models using *Yr17* and all markers with an increase of 0.17 and 0.16, respectively, for IT and 0.13 and 0.10, respectively, for SEV (Supplementary Tables 8, 9; Supplementary Figure 10). The validation sets were the only prediction scenarios in which MAS performed better than GS models. However, this was not the case for all MAS models, with most major markers showing similar decreases in the accuracy compared to cross-validations. Additionally, the RMSE was similar to cross-validations with low values for GS models compared to MAS across all markers (Supplementary Figure 11). Further, for SEV, an RMSE for MAS decreased with the addition of years.

#### *De novo* GWAS Markers

The *de novo* GWAS marker sets also increased the accuracy when more environments were included. The increase in the prediction accuracy was not seen in the previous validation sets as seen for the molecular markers for major rust genes. The *de novo* GWAS markers had the largest prediction accuracies in the last two validation sets with GWAS_5 having an accuracy of 0.33 for IT and GWAS having an accuracy of 0.38 for SEV using 2013–2017 to predict 2018 (Supplementary Tables 6, 7). In the last validation set, GWAS had the largest prediction accuracy of 0.55 for IT. Similarly, the smaller GWAS sets had the highest prediction accuracy. In contrast to the cross-validations, the larger GWAS sets did not have a drastic decrease with GWAS_100, and actually had the same prediction accuracy as the base rrBLUP for IT and an increase of 0.01 for SEV in using 2013–2018 to predict 2020 (Supplementary Tables 6, 7). The *de novo* GWAS marker sets had the largest increases in overall scenarios with GWAS_5 having an increase of 0.19 with MAS for IT (Supplementary Tables 8, 9; Supplementary Figure 12). Further, MAS for GWAS_100 displayed a much higher RMSE with the highest value for all scenarios reaching an RMSE of 381.71 using 2013–2015 to predict 2016 (Supplementary Figure 13; Supplementary Table 9). This prediction scenario was the only scenario using only the DP lines to predict BLs. However, all of the other GS-GWAS sets had an RMSE similar to the major markers for GS and MAS.

### Overall Differences

When comparing the different models over all years within each population, we found that the marker for *Yr17* and the combination of all markers had the largest prediction accuracies. However, the increase was only statistically significant in the BL population and in the validation sets. There was no statistical increase in the prediction accuracy in the DP. The largest mean accuracy in any population was the major rust markers and base rrBLUP for SEV in the DP with an accuracy of 0.64 across all years (Table 5). There was also a statistical increase in the prediction accuracy as we increased the combination of years over both IT and SEV training populations with the accuracies of 0.57 and 0.63 for IT and SEV, respectively when years 1–4 were combined (Table 6).

**Table 5:** Comparison of genomic selection models accuracy and pairwise comparisons for stripe rust IT and disease severity (SEV) for PNW winter wheat DP lines and BLs phenotyped from 2013 to 2020 in Central Ferry, Lind, and Pullman, WA, USA over all individual population cross-validation sets and combined validation sets.

**Trait**	**Pop**	**rrBLUP**	***Yr17***	***Yr10***	**IWB12603**	***Lr68***	**All_M**	**GWAS_B**	**GWAS_5**	**GWAS_10**	**GWAS_25**	**GWAS_50**	**GWAS_100**
IT	BL	0.58b	0.59a	0.58b	0.58b	0.58b	0.59a	0.57c	0.57c	0.56d	0.54e	0.52f	0.51g
	DP	0.53ab	0.53ab	0.53a	0.52b	0.53ab	0.52b	0.47c	0.47c	0.44d	0.42e	0.41f	0.4g
	VS	0.48d	0.49b	0.49cd	0.48d	0.48d	0.49ab	0.5a	0.49bc	0.48d	0.45e	0.44f	0.43g
SEV	BL	0.59b	0.6ab	0.59ab	0.59b	0.59b	0.6a	0.57cd	0.58c	0.56d	0.55e	0.53f	0.52g
	DP	0.64ab	0.64ab	0.64a	0.63b	0.64ab	0.64ab	0.6c	0.6c	0.57d	0.54e	0.54f	0.53g
	VS	0.54bc	0.55a	0.54abc	0.54abc	0.54abc	0.55ab	0.54cd	0.54d	0.54cd	0.52e	0.52e	0.52e

*Models labeled with the same letter are not significantly different (p = 0.05). Adjustments: ALL_M: IWB12603(Qyr.wpg-1B.1), KASP (Lr68), Xpsp3000(Yr10), and KASP (Yr17) combined; GWAS_B, genome-wide association assisted genomic selection (GWAS-GS) with Bonferonni significant markers; GWAS_5, GWAS-GS with the top 5 significant markers; GWAS_10, GWAS-GS with the top 10 significant markers; GWAS_25, GWAS-GS with the top 25 significant markers; GWAS_50, GWAS-GS with the top 50 significant markers; GWAS_100, GWAS-GS with the top 100 significant markers*.

**Table 6:** Comparison of the number of years in the training populations on overall GS model accuracy and pairwise comparisons for stripe rust infection type (IT) and disease severity (SEV) for PNW winter wheat over both the DP lines and BLs phenotyped from 2013 to 2020 in Central Ferry, Lind, and Pullman, WA, USA in the cross-validation sets.

**Trait**	**Year 1**	**Year 2**	**Year 3**	**Year 4**	**Year 1–2**	**Year 1–3**	**Year 1–4**
IT	0.52d	0.43f	0.57a	0.54c	0.49e	0.56b	0.57a
SEV	0.53f	0.55e	0.61b	0.59c	0.58d	0.62a	0.63a

*Models labeled with the same letter are not significantly different (P-value = 0.05)*.

## Discussion

### *GS* for Disease Resistance

The development of resistant cultivars is the most effective and economical method for controlling diseases such as stripe rust (Chen and Kang, 2017). Due to the challenges of breeding for both quantitative and qualitative disease resistance, it is recommended to combine them. In addition to the challenges for breeding both major gene qualitative disease resistance and minor gene quantitative resistance are also the common challenges of implementing and integrating any major gene or QTL into new cultivars. These difficulties include inconsistent effects of the QTL due to inconsistent QTL segregations in mapping populations, QTL interaction with genetic background, and QTL interaction with the environment (Bernardo, 2008). However, in addition to the common challenges, qualitative resistance also faces the disadvantage of new virulent races of a pathogen that can overcome major gene resistance (Chen and Kang, 2017). Breeding for minor gene quantitative resistance tends to produce a more durable resistance in BLs because it relies on multiple small-effect alleles. Similar to other agronomic traits, breeding for quantitative resistance requires multiple breeding cycles to gradually improve resistance (Poland and Rutkoski, 2016). The lack of qualitative resistance durability coupled with the challenge in identifying and breeding for quantitative resistance creates a unique opportunity for GS to identify quantitative resistance by accounting for minor-effect genes in the presence of large-effect major genes.

The goal of this study was to identify the best GS method for disease resistance in the presence of both major and minor genes. In our study, we used stripe rust as an example of the disease with both major and minor resistant genes. Previous studies on the GS of stripe rust showed promising prediction accuracies. Muleta et al. (2017) showed that accuracy increased with population size and marker density and reached up to 0.80. Ornella et al. (2012) reported accuracies in The International Maize and Wheat Improvement Center (CIMMYT) wheat populations of values greater than 0.50 for stripe rust, but showed a lower accuracy when compared to stem rust. In our study, the prediction accuracy for both IT and SEV reached an accuracy of up to 0.67 and 0.69 in cross-validations, respectively. Further, IT and SEV reached the accuracies of up to 0.66 and 0.72 in validation sets, respectively. In comparison to other rust diseases, Rutkoski et al. (2014, 2015) showed promising results to predict stem rust with the accuracies of up to 0.50. Overall, our study showed high prediction accuracies in comparison to most rust prediction studies, and further displayed the feasibility for accurately predicting disease resistance in the presence of major and minor resistant genes.

### Major Markers

When major genes are present, a large portion of the genetic variance for a trait may be due to the unknown QTL with minor effects (Bernardo, 2014). The other minor-effect QTL will not necessarily be integrated when major genes are integrated into cultivars. The lack of integration can be attributed to not being able to use MAS and the difficulties outlined previously in pyramiding major-effect genes. In contrast, GS simultaneously models all QTL (Meuwissen et al., 2001). However, the use of GS models such as rrBLUP will underestimate the effect of the major QTL. Therefore, the inclusion of the major-effect QTL as fixed effects can increase accuracy. According to Bernardo (2014), major genes should be used in prediction models when only a few major genes are present, and each gene accounts for more than 10% of the variation.

In this study, the major gene in both populations was *Yr17*. In the BL, *Yr17* accounted for up to 0.40 prediction accuracy when used in MAS, and therefore accounts for a large amount of variation. The moderate accuracy of *Yr17* supports that even with the degradation of the ASR for *Yr17*, it still provides resistance for APR as indicated in Liu et al. (2018). The other major rust genes present in the BL would be considered as minor-effect genes with a near-zero prediction accuracy within MAS or, in the case of the marker for *Yr10*, only produced an accuracy greater than 0.10 in a few prediction scenarios. The higher accuracy in the BL for Yr17 also shows a lower RMSE compared to the other markers. However, within the DP, all of the markers with the exception of *Lr68* produced accuracies greater than 0.20, with *IWB12603* reaching 0.34 and *Yr10* reaching the highest accuracies for MAS within cross-validations of 0.42, and could be considered as major-effect markers. Additionally, the higher accuracy for Yr10 was coupled with a lower RMSE than the other markers.

Even with the moderate accuracies of the major rust markers in MAS, we observed only a slight increase in the prediction accuracy when the major markers were included in our GS models, and relatively a lower RMSE than the MAS. The major markers only increased the prediction accuracy at a maximum of 0.06 within the cross-validation scenarios and 0.03 within the validation sets. Interestingly, the validation sets resulted in the highest accuracy of all scenarios with 0.72 for the base GS model and the inclusion of the major markers when predicting SEV in 2014 using 2013 with the small RMSE values for all markers. These results are in direct contrast to previous studies showing a higher accuracy in cross-validations (Lozada and Carter, 2019; Merrick and Carter, 2021). Validation sets are a more realistic approach for GS because it is comparable to how GS would be implemented in breeding programs (Lozada and Carter, 2019). However, the major markers only increased the prediction accuracy as the overall prediction accuracy decreased. For example, using 2013–2017 to predict 2018, all of the major markers increased the prediction accuracy, but the base prediction was only 0.27, and the markers increased the accuracy by 0.03 maximum in all scenarios. Further, the major markers had much larger increases in the MAS scenarios with a maximum increase of 0.17, but resulted in a higher RMSE. Therefore, the inclusion of the major markers provides an advantage in the more realistic validation sets when the base GS model has poor predictive ability.

In the context of GS models and breeding programs, a small increase in the prediction accuracy would be considered negligible in realistic breeding scenarios. The results in our study are in contrast to previous studies showing that the major markers had a large increase in prediction accuracies in GS models for other diseases such as stem rust (Rutkoski et al., 2014) and Fusarium head blight (Arruda et al., 2016). One hypothesis for the lack of increase in the prediction accuracy may be due to GS models accounting for a majority of variation in both the major- and minor-effect markers for disease resistance, or the major-effect markers may be accounted for in the models of the GBS markers. However, the ridge regression penalty reduces the effect of large-effect markers, hence the additional variation would need to be accounted for by other small-effect markers (Rice and Lipka, 2019). Additionally, the lack of increase in the prediction accuracy may be due to the major markers not accounting for enough phenotypic variation. Due to the reduction in the effect of the major markers, Bernardo (2014) suggested implementing markers that account for more than 10% of the variation as mentioned previously. This theory may be disproved by the major markers that display a moderate accuracy in MAS models. However, this may be the case for *Lr68*, which displayed a minimal effect in both MAS models and GS models.

Further, the lack of increase in the prediction accuracy may be beneficial in demonstrating that other uncharacterized resistant QTL can still provide a large amount of disease resistance within the populations either alone or in conjunction with major genes. In this case, our results would be beneficial in confirming the presence of minor-effect QTL for quantitative resistance and provide a more durable resistance within the training populations. Therefore, we can conclude that genotyping and selecting major genes for disease resistance may not be necessary when the breeding programs can use more cost-effective genome-wide markers to implement GS with more consistent results.

### *De novo* GWAS Markers

Frequently, the major markers for disease resistance are either unknown or have an uncharacterized effect within the populations. Therefore, GWAS can be performed to characterize disease-resistant QTL within a population, and the significant markers can be used as fixed-effect covariates (Rice and Lipka, 2019). In Zhang et al. (2014), publicly available GWAS markers were integrated into prediction models but only increased the accuracy by 0.01, similar to our results. In contrast, we used *de novo* GWAS markers dependent on the training population. This approach has been used for FHB in which Arruda et al. (2016) demonstrated an increase in the accuracy of up to 0.14. These results were also demonstrated in Spindel et al. (2016), in which *de novo* GWAS markers implemented into GS increased the accuracies more than 0.10 in rice (*Oryza sativa* L.). However, in our study, the *de novo* GWAS markers only marginally increased the accuracy, or in the case of implementing more than 25 markers, decreased accuracy in the majority of cross-validation scenarios and an increased RMSE. A reduction in the prediction accuracy and an increase of RMSE with a larger set of *de novo* GWAS markers may be attributed to an increase in the bias of the model and an increase of RMSE due to overfitting as seen in Raymond et al. (2018) or due to the difficulty experienced by the model to simultaneously estimate all of the fixed effects (Bernardo, 2014). A reduction in the prediction accuracy was also shown in Rice and Lipka (2019).

Another hypothesis may be stated for why the *de novo* GWAS markers failed to increase the prediction accuracy due to the inclusion of false positives within GWAS models. To mitigate this, we included a GWAS-GS model that only included significant markers based on a Bonferroni correction of 0.05. However, this model failed to self-differentiate from another smaller set of GWAS-GS models. The lack of reduction was mainly seen in our cross-validation sets. Within cross-validation, the training population is divided. The division of the training populations may be one cause of the lack of increase of the prediction accuracy. The smaller validation fold within a cross-validation may have a weak association with the markers found in the larger training folds, as hinted at by Rice and Lipka (2019). The weak association theory may be supported by the contrasting results seen in the validation sets.

Similar to the inclusion of the major markers in the cross-validations, the validation sets showed an increase in the prediction accuracy when the *de novo* GWAS markers were included and displayed the largest increases from GS models. The GWAS model with significant markers only (GWAS_B) displayed the largest increase of 0.06 in the SEV. Once again, this increased prediction accuracy was observed as the prediction accuracy of the base GS model decreased. This occurrence in both the major and *de novo* GWAS markers demonstrates the ability to increase and maintain a high accuracy as the GS model fails in predicting lines. Therefore, we can conclude that even though fixed-effect markers may not increase the accuracy in typical cross-validation scenarios, they are beneficial in more realistic validation set approaches similar to the major markers.

However, similar to the major markers, increased prediction accuracy with the inclusion of *de novo* GWAS markers was very small relative to the high accuracy for most scenarios. Further, small sets of *de novo* GWAS markers were similar in consistency to the major markers. Therefore, there is little benefit in characterizing major-effect disease resistance markers for GS over implementing the GWAS-GS methods that would use the same sets of markers like GS models.

### Training Population and Environment

We compared the effect of the major and *de novo* GWAS markers in different training populations that are commonly used in breeding programs. The frequency and source of both major disease- and minor disease-resistant genes vary. For instance, the BL population consists of WSU BLs that have been selected for resistance, specifically for *P. striiformis* f. sp. *tritici* races in Washington, and therefore has a high level of resistance throughout the population. In comparison, the DP consists of varieties from various breeding programs in the PNW. The sources of resistance in the varieties are more similar within the BL than in the DP, with the DP containing major genes different from the major markers chosen in this study common in the WSU germplasm or selected for resistance to races not present in eastern Washington.

The differences in the frequency of major genes were observed in the major rust markers used in this study. In the BL, the *Yr17* marker showed an increase in the prediction accuracy for GS models and a relatively high accuracy in MAS models compared to the other markers. However, this was not consistently seen in the DP. The inconsistent effect of *Yr17* in different training populations may be due to the higher frequency of *Yr17* in the BL compared to the DP. This may also be supported by the higher accuracies for *Yr10* and *IWB12603* in the DP compared to the BL, and both of these rust genes have a higher frequency in the DP than in the BL. Our study showed that regardless of the frequency of the rust-resistant genotypes, there was only a small to nil increase in the prediction accuracy. Therefore, GS would be more accurate than MAS regardless of the frequency of the known rust-resistant genotypes in a breeding program due to the ability to account for both major disease and minor disease-resistant genes.

In addition to different frequencies of major genes, the general composition of the training populations can affect GS prediction accuracy (Asoro et al., 2011). The composition of the training population affects the accuracy due to both population structure and genetic relatedness (Habier et al., 2007; Asoro et al., 2011; Mirdita et al., 2015). We compared the population structure in our models by plotting principal components and identified three clusters indicating distinct subpopulations. In addition, the population structure was not taken into account in our GS models. However, we can see the effect of genetic relatedness and population in both our cross-validation and validation sets. The BL had a statistically higher mean accuracy for both IT and SEV than the DP in cross-validations, which could be attributed to the closer genetic relatedness of the population and sources of resistance as mentioned previously. A higher prediction accuracy for the BL is advantageous for breeding programs because they can use their existing breeding trials for GS without screening a DP outside their breeding program. In the validation sets, we see an initial increase in the accuracy due to the DP being the only population in the training populations, but as we added in BLs, the accuracy decreased. The accuracy was reduced when the DP predicted the BL, but eventually increased as more BLs were introduced into the training population. The decrease in validation sets can also be attributed to GEI (Michel et al., 2016; Huang et al., 2018; Lozada and Carter, 2019, 2020; Haile et al., 2020).

Further, GEI is important for qualitative disease resistance. Race-specific qualitative resistance is dependent on the race in the environment and thus can lead to larger environmental effects (Poland and Rutkoski, 2016). In contrast, GEI has a much smaller effect on minor-gene quantitative resistance due to the lack of a gene-for-gene interaction. In our study, the most frequent races were similar from year to year, and therefore may not be a significant factor in the differing prediction accuracy.

In this study, disease resistance screening was dependent on the natural occurrence of stripe rust for disease pressure, and therefore the overall effect of the environment is important. Additionally, diseases such as stripe rust are affected by several environmental factors, including moisture, temperature, and wind. Further, disease SEV is affected by the other aspects of the disease triangle, disease inoculum, and a susceptible host to induce disease development (Chen, 2005). Disease development and the quality of the phenotypic data obtained from the unreplicated trials may also explain the differences in the prediction accuracy from year to year, especially in the DP in which the same lines are phenotyped every year. Meanwhile, BLs are only phenotyped in a single year, and therefore the difference from 1 year to the next can be either disease incidence as in the DP or the changes occurred due to differing levels of resistance within BLs. In addition, we see an increase in the prediction accuracy for both the cross-validation and validation sets as we increase the number of environments within our training population. The increase in accuracy may be accounted for by the inclusion of GEI within our phenotypic adjustments and GS models as reported in previous studies, as well as the general high heritability for disease resistance (Crossa et al., 2014; Jarquín et al., 2014; Haile et al., 2020; Merrick and Carter, 2021). Overall, our GS models accurately predicted disease resistance in different training populations and environments, and therefore will be an important strategy for selecting for disease resistance.

### Applications in Breeding

Genome selection is beneficial for complex traits and can outperform phenotypic selection and MAS for low heritable traits. However, there may be little benefit in using GS for selection purposes for highly heritable traits such as disease resistance (Poland and Rutkoski, 2016). In the case of highly heritable traits, GS can still outperform phenotypic selection and MAS in terms of gain per unit time when implemented in the early stages of the breeding cycle (Bernardo and Yu, 2007; Rutkoski et al., 2011). In our study, a high prediction accuracy would allow an increase in genetic gain by decreasing the cycle time of the breeding program and rapidly accumulating favorable alleles for disease resistance (Rutkoski et al., 2011).

Even though phenotypic selection has been successfully implemented for disease resistance, without controlled experiments, one cannot determine whether the resistance is quantitative or qualitative. Therefore, we cannot conclude whether the resistance will be durable in the long term. Alternatively, we can implement MAS to select qualitative and quantitative disease resistance within the BLs to bypass the need for controlled experiments. However, as seen in our study, MAS does not account for all of the resistance within the lines in either of the training populations, as shown by a decrease in the prediction accuracy for MAS models. MAS also has limitations when it comes to pyramiding multiple markers, as discussed previously, and is a form of tandem selection (Bernardo, 2014). In contrast, GS is a form of selection index and has been shown to be superior to tandem selection (Hazel and Lush, 1942). Using GS, we can select for the accumulation of all-resistant QTL to take advantage of the quantitative and qualitative resistant genes within a population, even when they are uncharacterized. Furthermore, by using fixed effects, we can select the lines that have a major marker of interest (Poland and Rutkoski, 2016). Therefore, GS will have a place in selecting for both quantitative and qualitative disease resistance.

Another advantage in implementing GS is by reducing both genotyping and phenotyping within a breeding program. GS can remove the need for genotyping for major and minor genes for selection purposes. This is further supported by the similar accuracies between major and *de novo* GWAS markers. By utilizing genome-wide markers, we can not only implement GS or GWAS-GS but also utilize the markers for additional traits, thus making the genome-wide markers more cost-effective (Poland and Rutkoski, 2016). Likewise, with the help of GS, breeding programs can reduce the need for phenotypic screening in disease nurseries in multiple locations and free up resources for screening more lines and increase genetic gain (Poland and Rutkoski, 2016).

Furthermore, the challenges introduced by the environment mentioned previously provide another advantage in using GS for disease resistance. GS models will help select cultivars with durable quantitative resistance with the accumulation of favorable alleles and select for disease resistance in environments not conducive to disease incidence needed for phenotypic selection. Overall, the high accuracy of GS models in our study displays the ability to predict durable disease resistance and account for uncharacterized minor-effect QTL in the presence of known major genes.

## Conclusions

This study showed the ability to accurately predict disease resistance using major and minor genes. The small to nil increase in the prediction accuracy for the major markers indicates the need for a careful selection of the major markers that account for a large variation in the training and test populations. Further, a comparison of the number of *de novo* GWAS markers shows that a small number of *de novo* GWAS markers should be used instead of a large set of markers to keep from overfitting the model. Additionally, fixed-effect markers may not provide a benefit in scenarios with already high prediction accuracy. However, in prediction scenarios with low accuracies such as in more realistic validation sets, the inclusion of both major markers and *de novo* GWAS helps to account for a variation in case of the failure of the base GS models. Moreover, we can increase the accuracy with the inclusion of additional environments and by using the populations that are genetically related such as the BL. Overall, there were no disadvantages in the inclusion of the major or *de novo* GWAS markers. The lack of increase of the prediction accuracy with the inclusion of fixed effects coupled with a large decrease in the accuracy using MAS indicates the presence of minor-effect QTL for quantitative resistance and thus durable resistance within the training populations. This study showed the ability to predict disease resistance and accumulate favorable alleles for durable disease resistance in the presence of major and minor resistance genes.

## Data Availability Statement

The datasets presented in this study can be found in online repositories. The names of the repository/repositories and accession number(s) can be found at: https://github.com/lfmerrick21/Major-and-Minor-Genes.

## Author Contributions

LM conceptualized the idea, analyzed the data, and drafted the manuscript. AB genotyped the KASP markers, reviewed, and edited the manuscript. XC reviewed and edited the manuscript. AC supervised the study, conducted field trials, edited the manuscript, and obtained the funding for the project. All authors contributed to the article and approved the submitted version.

## Conflict of Interest

The authors declare that the research was conducted in the absence of any commercial or financial relationships that could be construed as a potential conflict of interest.

## Publisher's Note

All claims expressed in this article are solely those of the authors and do not necessarily represent those of their affiliated organizations, or those of the publisher, the editors and the reviewers. Any product that may be evaluated in this article, or claim that may be made by its manufacturer, is not guaranteed or endorsed by the publisher.
