# Supplementary material for: Breeding With Major and Minor Genes: Genomic Selection for Quantitative Disease Resistance
Source: Front Plant Sci. 2021 Aug 6;12:713667. doi: 10.3389/fpls.2021.713667 (PMC8377761; doi:10.3389/fpls.2021.713667)
Supplement: Supplementary file 1 [file Data_Sheet_1.docx]

| **Table S1.** Stripe rust race summary and major race frequency across Eastern Washington in 2013 to 2020. | | | | |
| --- | --- | --- | --- | --- |
| Year | No. of Isolates | No. of races | Major Races | Freqency (%) |
| 2020 | 158 | 14 | PSTv-37 | 50.6 |
|  |  |  | PSTv-39 | 22.8 |
| 2018 | 150 | 20 | PSTv-37 | 38.7 |
|  |  |  | PSTv-52 | 10.7 |
|  |  |  | PSTv-322 | 8 |
| 2017 | 104 | 43 | PSTv-37 | 40.4 |
|  |  |  | PSTv-322 | 4.8 |
| 2016 | 149 | 37 | PSTv-37 | 19.5 |
|  |  |  | PSTv-52 | 13.4 |
| 2015 | 130 | 23 | PSTv-37 | 30 |
|  |  |  | PSTv-52 | 23.8 |
| 2014 | 129 | 22 | PSTv-37 | 10.1 |
|  |  |  | PSTv-48 | 10.9 |
|  |  |  | PSTv-52 | 25.6 |
|  |  |  | PSTv-79 | 14.7 |
| 2013 | 180 | 26 | PSTv-11 | 10 |
|  |  |  | PSTv-37 | 12.8 |
|  |  |  | PSTv-52 | 26.7 |
|  |  |  | PSTv-73 | 9.4 |
|  | | | | |

| **Table S2.** Comparison of genomic selection models accuracy, root-mean square error, and pairwise comparisons for stripe rust infection type for Pacific Northwest winter wheat diversity panel (DP) lines and breeding lines (BL) phenotyped from 2013-2020 in Central Ferry, Lind, and Pullman, WA using cross-validation within and across trials. | | | | | | | | | | | | | |
| --- | --- | --- | --- | --- | --- | --- | --- | --- | --- | --- | --- | --- | --- |
| Population | Year | rrBLUP | IWB12603 | *Lr68* | *Yr10* | *Yr17* | All_M | GWAS_B | GWAS_5 | GWAS_10 | GWAS_25 | GWAS_50 | GWAS_100 |
| BL | 2016 | 0.57(3.74)cd* | 0.6(3.65)ab | 0.58(3.69)bc | 0.57(3.74)cd | 0.56(3.77)d | 0.61(3.62)a | 0.53(3.92)e | 0.5(4.03)f | 0.48(4.15)g | 0.46(4.3)h | 0.45(4.42)h | 0.42(4.63)i |
| BL | 2017 | 0.48(3.68)bc | 0.49(3.64)a | 0.47(3.69)cd | 0.48(3.68)bc | 0.48(3.67)bc | 0.48(3.66)ab | 0.46(3.73)d | 0.46(3.73)d | 0.43(3.85)e | 0.4(3.97)f | 0.39(4.06)fg | 0.38(4.16)g |
| BL | 2018 | 0.65(4.35)cd | 0.65(4.34)bcd | 0.65(4.34)cd | 0.65(4.35)cd | 0.65(4.36)d | 0.66(4.33)bcd | 0.66(4.32)b | 0.67(4.27)a | 0.66(4.34)bc | 0.63(4.5)e | 0.62(4.59)f | 0.6(4.75)g |
| BL | 2020 | 0.65(4.68)bc | 0.66(4.61)ab | 0.66(4.64)ab | 0.66(4.66)ab | 0.67(4.6)ab | 0.67(4.58)a | 0.66(4.68)ab | 0.66(4.65)ab | 0.64(4.83)c | 0.61(5.07)d | 0.59(5.26)e | 0.56(5.57)f |
| BL | 2016-2017 | 0.51(3.7)b | 0.52(3.66)a | 0.51(3.71)b | 0.51(3.71)b | 0.51(3.71)b | 0.52(3.67)a | 0.49(3.75)c | 0.49(3.75)c | 0.47(3.82)d | 0.45(3.93)e | 0.43(4.02)f | 0.43(4.1)f |
| BL | 2016-2018 | 0.6(4.1)c | 0.61(4.08)ab | 0.6(4.1)c | 0.6(4.1)c | 0.61(4.1)c | 0.61(4.08)ab | 0.61(4.1)bc | 0.61(4.06)a | 0.61(4.07)a | 0.6(4.15)d | 0.58(4.24)e | 0.56(4.32)f |
| BL | 2016-2020 | 0.61(4.22)e | 0.62(4.19)bc | 0.61(4.21)de | 0.61(4.21)de | 0.61(4.21)e | 0.61(4.19)bc | 0.61(4.21)cd | 0.62(4.17)a | 0.62(4.18)ab | 0.6(4.26)f | 0.59(4.32)g | 0.58(4.41)h |
| DP | 2013 | 0.55(1.86)a | 0.55(1.87)a | 0.55(1.86)a | 0.55(1.86)a | 0.55(1.87)a | 0.55(1.86)a | 0.47(1.99)b | 0.48(1.99)b | 0.45(2.05)c | 0.43(2.11)d | 0.4(2.18)e | 0.41(2.21)e |
| DP | 2014 | 0.46(2.69)a | 0.45(2.7)a | 0.46(2.69)a | 0.45(2.7)a | 0.45(2.71)a | 0.44(2.72)a | 0.4(2.83)b | 0.39(2.84)b | 0.35(2.97)cd | 0.34(3.06)cd | 0.35(3.06)c | 0.34(3.14)d |
| DP | 2015 | 0.54(2.36)a | 0.54(2.36)a | 0.55(2.35)a | 0.54(2.36)a | 0.54(2.37)a | 0.54(2.36)a | 0.49(2.49)b | 0.48(2.5)b | 0.45(2.58)c | 0.43(2.67)d | 0.41(2.73)d | 0.39(2.83)e |
| DP | 2016 | 0.5(3.35)a | 0.49(3.35)a | 0.5(3.34)a | 0.5(3.34)a | 0.49(3.36)a | 0.49(3.37)a | 0.41(3.59)b | 0.41(3.59)b | 0.36(3.76)c | 0.35(3.88)cd | 0.34(3.98)de | 0.33(4.06)e |
| DP | 2013-2014 | 0.54(1.89)a | 0.54(1.89)a | 0.55(1.89)a | 0.54(1.9)a | 0.54(1.9)a | 0.54(1.9)a | 0.47(2.02)b | 0.48(2)b | 0.44(2.1)c | 0.42(2.16)cd | 0.41(2.2)de | 0.4(2.25)e |
| DP | 2013-2015 | 0.54(1.8)a | 0.55(1.8)a | 0.55(1.8)a | 0.54(1.8)a | 0.54(1.81)a | 0.54(1.81)a | 0.52(1.87)b | 0.51(1.87)b | 0.5(1.92)c | 0.47(2)d | 0.47(2.01)d | 0.46(2.07)d |
| DP | 2013-2016 | 0.56(1.78)a | 0.56(1.79)a | 0.57(1.78)a | 0.57(1.78)a | 0.56(1.79)a | 0.56(1.79)a | 0.54(1.85)b | 0.53(1.85)b | 0.51(1.9)c | 0.49(1.99)d | 0.48(2.02)de | 0.47(2.06)e |
| *Models labeled with the same letter are not significantly different (P-value =0.05);Numbers in parenthesis are root-mean square error of prediction accuracy.  Adjustments: ALL_M: IWB12603(Qyr.wpg-1B.1), KASP(Lr68), Xpsp3000(Yr10), and KASP(Yr17) combined; GWAS_B: genome-wide association assisted genomic selection (GWAS-GS) with Bonferonni significant markers; GWAS_5: GWAS-GS with the top 5 significant markers; GWAS_10: GWAS-GS with the top 10 significant markers; GWAS_25: GWAS-GS with the top 25 significant markers; GWAS_50: GWAS-GS with the top 50 significant markers; and GWAS_100: GWAS-GS with the top 100 significant markers. | | | | | | | | | | | | | |

| **Table S3.** Comparison of genomic selection models accuracy, root-mean square error, and pairwise comparisons for stripe rust disease severity for Pacific Northwest winter wheat diversity panel (DP) lines and breeding lines (BL) phenotyped from 2013-2020 in Central Ferry, Lind, and Pullman, WA using cross-validation within and across trials. | | | | | | | | | | | | | |
| --- | --- | --- | --- | --- | --- | --- | --- | --- | --- | --- | --- | --- | --- |
| Population | Year | rrBLUP | IWB12603 | *Lr68* | *Yr10* | *Yr17* | All_M | GWAS_B | GWAS_5 | GWAS_10 | GWAS_25 | GWAS_50 | GWAS_100 |
| BL | 2016 | 0.51(20.63)c* | 0.54(20.36)bc | 0.55(20.14)b | 0.52(20.55)c | 0.53(20.51)bc | 0.58(19.77)a | 0.4(23.92)d | 0.4(23.77)d | 0.38(24.75)d | 0.35(26.88)e | 0.34(27.46)ef | 0.32(30.88)f |
| BL | 2017 | 0.5(27.85)bc | 0.5(27.71)abc | 0.5(27.88)cd | 0.5(27.84)cd | 0.5(27.86)c | 0.5(27.76)abc | 0.51(27.77)a | 0.51(27.8)ab | 0.49(28.41)d | 0.47(29.22)e | 0.45(29.97)f | 0.44(30.71)g |
| BL | 2018 | 0.68(34.16)bc | 0.68(34.12)bc | 0.68(34.09)bc | 0.68(34.09)bc | 0.68(34.16)bc | 0.68(34.16)bc | 0.68(34.02)b | 0.69(33.52)a | 0.67(34.38)c | 0.66(35.71)d | 0.64(36.15)e | 0.63(37.36)f |
| BL | 2020 | 0.66(33.62)ab | 0.67(33.39)a | 0.67(33.45)a | 0.66(33.57)ab | 0.67(33.35)a | 0.66(33.57)ab | 0.64(35.27)cd | 0.65(34.5)bc | 0.63(35.68)d | 0.61(36.98)e | 0.6(38.26)f | 0.57(41.26)g |
| BL | 2016-2017 | 0.51(25.79)bc | 0.52(25.69)ab | 0.52(25.7)ab | 0.52(25.76)abc | 0.51(25.8)bc | 0.53(25.61)a | 0.51(26.01)cd | 0.51(25.9)bc | 0.5(26.18)d | 0.48(26.99)e | 0.46(27.61)f | 0.45(28.2)g |
| BL | 2016-2018 | 0.63(31.08)c | 0.63(31)c | 0.63(31.09)c | 0.63(31.09)c | 0.63(31.07)c | 0.63(30.99)c | 0.64(30.79)b | 0.64(30.68)ab | 0.64(30.56)a | 0.63(31.07)c | 0.62(31.61)d | 0.6(32.29)e |
| BL | 2016-2020 | 0.63(31.69)bcd | 0.63(31.61)b | 0.63(31.68)bcd | 0.63(31.7)bcd | 0.63(31.74)cd | 0.63(31.62)bc | 0.63(31.49)a | 0.64(31.4)a | 0.64(31.36)a | 0.62(31.88)d | 0.61(32.5)e | 0.6(33.11)f |
| DP | 2013 | 0.64(23)a | 0.64(23.05)a | 0.65(22.79)a | 0.64(22.96)a | 0.64(23.04)a | 0.64(22.92)a | 0.59(24.34)b | 0.59(24.25)b | 0.56(25.18)c | 0.53(26.31)d | 0.52(26.96)e | 0.52(27.33)e |
| DP | 2014 | 0.63(26.69)a | 0.63(26.67)a | 0.63(26.68)a | 0.63(26.63)a | 0.63(26.77)a | 0.63(26.76)a | 0.59(28.14)b | 0.61(27.71)b | 0.58(28.9)c | 0.55(29.93)d | 0.54(30.59)de | 0.53(31.32)e |
| DP | 2015 | 0.6(33.91)ab | 0.59(33.93)ab | 0.6(33.59)a | 0.6(33.79)ab | 0.59(34.05)b | 0.6(33.78)ab | 0.56(34.98)c | 0.55(35.56)d | 0.51(36.94)e | 0.48(38.57)f | 0.47(39.42)g | 0.46(40.2)g |
| DP | 2016 | 0.58(41.26)a | 0.58(41.23)a | 0.58(41.35)a | 0.58(41.1)a | 0.57(41.4)a | 0.57(41.4)a | 0.55(42.52)b | 0.54(42.96)b | 0.51(44.73)c | 0.49(46.39)d | 0.48(47.25)de | 0.47(48.1)e |
| DP | 2013-2014 | 0.69(20.97)a | 0.68(21.07)a | 0.69(21.08)a | 0.68(21.09)a | 0.68(21.13)a | 0.69(21.04)a | 0.65(22.14)c | 0.67(21.72)b | 0.63(22.84)d | 0.61(23.83)e | 0.6(24.26)e | 0.58(24.93)f |
| DP | 2013-2015 | 0.65(23.05)a | 0.65(23.16)a | 0.66(23)a | 0.65(23.07)a | 0.65(23.13)a | 0.65(23.14)a | 0.61(24.29)b | 0.61(24.21)b | 0.59(25.07)c | 0.57(25.83)d | 0.57(26.09)d | 0.56(26.89)e |
| DP | 2013-2016 | 0.67(22.79)a | 0.67(22.81)a | 0.67(22.8)a | 0.67(22.78)a | 0.67(22.81)a | 0.67(22.84)a | 0.63(24.14)c | 0.64(23.78)b | 0.61(24.74)d | 0.58(26.01)e | 0.58(26.26)ef | 0.57(26.85)f |
| *Models labeled with the same letter are not significantly different (*P*-value =0.05); Numbers in parenthesis are root-mean square error of prediction accuracy.  Adjustments: ALL_M: IWB12603(Qyr.wpg-1B.1), KASP(Lr68), Xpsp3000(Yr10), and KASP(Yr17) combined; GWAS_B: genome-wide association assisted genomic selection (GWAS-GS) with Bonferonni significant markers; GWAS_5: GWAS-GS with the top 5 significant markers; GWAS_10: GWAS-GS with the top 10 significant markers; GWAS_25: GWAS-GS with the top 25 significant markers; GWAS_50: GWAS-GS with the top 50 significant markers; and GWAS_100: GWAS-GS with the top 100 significant markers. | | | | | | | | | | | | | |

| **Table S4.** Comparison of marker-assisted selection models accuracy, root-mean square error, and pairwise comparisons for stripe rust infection type for Pacific Northwest winter wheat diversity panel (DP) lines and breeding lines (BL) phenotyped from 2013-2020 in Central Ferry, Lind, and Pullman, WA using cross-validation within and across trials. | | | | | | | | | | | | | |
| --- | --- | --- | --- | --- | --- | --- | --- | --- | --- | --- | --- | --- | --- |
| Population | Year | IWB12603 | *Lr68* | *Yr10* | *Yr17* | All_M | GWAS_B | GWAS_5 | GWAS_10 | GWAS_25 | GWAS_50 | GWAS_100 |  |
| BL | 2016 | 0.4(4.18)ab* | -0.01(4.56)d | 0.03(4.56)c | 0.05(4.56)c | 0.4(4.19)ab | 0.39(4.27)ab | 0.38(4.34)b | 0.41(4.35)a | 0.41(4.43)a | 0.41(4.54)a | 0.41(4.64)a |  |
| BL | 2017 | 0.21(4.1)c | 0.15(4.14)d | 0.15(4.14)d | 0.06(4.18)e | 0.29(4.01)b | 0.36(3.92)a | 0.37(3.92)a | 0.38(3.95)a | 0.36(4.05)a | 0.37(4.1)a | 0.37(4.18)a |  |
| BL | 2018 | 0.45(5.14)e | 0.07(5.73)g | -0.03(5.74)h | 0.09(5.72)f | 0.45(5.13)e | 0.6(4.6)a | 0.59(4.61)bc | 0.6(4.59)a | 0.6(4.65)ab | 0.59(4.75)cd | 0.58(4.84)d |  |
| BL | 2020 | 0.25(5.99)d | 0.08(6.16)ef | 0.07(6.17)f | 0.1(6.15)e | 0.24(6)d | 0.45(5.52)c | 0.46(5.52)c | 0.5(5.45)b | 0.52(5.5)ab | 0.53(5.54)a | 0.52(5.82)a |  |
| BL | 2016-2017 | 0.27(4.14)d | 0.12(4.27)e | 0.08(4.29)f | 0.04(4.3)g | 0.3(4.1)c | 0.36(4.03)b | 0.37(4.03)b | 0.39(4.02)a | 0.39(4.07)a | 0.39(4.14)a | 0.39(4.2)a |  |
| BL | 2016-2018 | 0.38(4.77)f | 0.08(5.13)g | 0.03(5.14)i | 0.04(5.14)h | 0.39(4.75)e | 0.51(4.43)b | 0.48(4.53)d | 0.51(4.45)c | 0.54(4.37)a | 0.54(4.4)a | 0.54(4.44)a |  |
| BL | 2016-2020 | 0.36(4.96)g | 0.08(5.3)h | 0.02(5.31)i | 0.01(5.32)j | 0.37(4.93)f | 0.52(4.54)c | 0.46(4.71)e | 0.51(4.58)d | 0.54(4.5)b | 0.55(4.48)a | 0.55(4.52)a |  |
| DP | 2013 | 0.19(2.19)g | 0.38(2.06)b | 0.08(2.22)h | 0.31(2.12)d | 0.42(2.02)a | 0.19(2.24)g | 0.23(2.23)f | 0.27(2.24)e | 0.32(2.23)d | 0.35(2.25)c | 0.35(2.29)c |  |
| DP | 2014 | 0.21(2.96)d | 0.23(2.94)c | -0.07(3.03)g | 0.14(2.99)f | 0.28(2.9)b | 0.19(3.08)e | 0.2(3.05)de | 0.25(3.09)c | 0.28(3.14)b | 0.3(3.18)ab | 0.31(3.19)a |  |
| DP | 2015 | 0.16(2.77)f | 0.42(2.55)b | 0.08(2.8)g | 0.33(2.65)d | 0.44(2.52)a | 0.3(2.73)e | 0.33(2.71)d | 0.37(2.69)c | 0.38(2.73)c | 0.39(2.76)c | 0.38(2.85)c |  |
| DP | 2016 | 0.19(3.8)e | 0.32(3.65)a | 0.01(3.86)g | 0.21(3.77)d | 0.35(3.62)a | 0.15(3.98)f | 0.18(3.94)e | 0.23(4.01)d | 0.26(4.06)c | 0.29(4.07)b | 0.29(4.15)b |  |
| DP | 2013-2014 | 0.21(2.21)ef | 0.34(2.12)b | 0.01(2.25)g | 0.23(2.2)e | 0.38(2.09)a | 0.21(2.27)ef | 0.2(2.27)f | 0.26(2.28)d | 0.31(2.29)c | 0.36(2.27)ab | 0.36(2.31)ab |  |
| DP | 2013-2015 | 0.15(2.13)g | 0.4(1.98)d | 0.09(2.15)h | 0.33(2.03)f | 0.42(1.95)c | 0.37(2.03)e | 0.38(2.01)de | 0.42(2.01)c | 0.43(2.06)bc | 0.44(2.06)ab | 0.44(2.09)a |  |
| DP | 2013-2016 | 0.16(2.14)f | 0.4(1.98)c | 0.08(2.15)g | 0.34(2.03)e | 0.43(1.95)ab | 0.38(2.02)d | 0.38(2.02)d | 0.42(2.03)bc | 0.44(2.05)a | 0.44(2.07)a | 0.45(2.1)a |  |
| *Models labeled with the same letter are not significantly different (*P*-value =0.05); Numbers in parenthesis are root-mean square error of prediction accuracy.  Adjustments: ALL_M: IWB12603(Qyr.wpg-1B.1), KASP(Lr68), Xpsp3000(Yr10), and KASP(Yr17) combined; GWAS_B: genome-wide association assisted genomic selection (GWAS-GS) with Bonferonni significant markers; GWAS_5: GWAS-GS with the top 5 significant markers; GWAS_10: GWAS-GS with the top 10 significant markers; GWAS_25: GWAS-GS with the top 25 significant markers; GWAS_50: GWAS-GS with the top 50 significant markers; and GWAS_100: GWAS-GS with the top 100 significant markers. | | | | | | | | | | | | | |

| **Table S5.** Comparison of marker-assisted selection models accuracy, root-mean square error, and pairwise comparisons for stripe rust disease severity for Pacific Northwest winter wheat diversity panel (DP) lines and breeding lines (BL) phenotyped from 2013-2020 in Central Ferry, Lind, and Pullman, WA using cross-validation within and across trials. | | | | | | | | | | | | | |
| --- | --- | --- | --- | --- | --- | --- | --- | --- | --- | --- | --- | --- | --- |
| Population | Year | IWB12603 | *Lr68* | *Yr10* | *Yr17* | All_M | GWAS_B | GWAS_5 | GWAS_10 | GWAS_25 | GWAS_50 | GWAS_100 |  |
| BL | 2016 | 0.42(21.75)a* | -0.09(23.88)e | -0.04(23.83)d | 0.17(23.75)c | 0.43(21.6)a | 0.24(28.27)b | 0.22(27.9)b | 0.23(29.82)b | 0.25(29.88)b | 0.24(30.74)b | 0.24(31.43)b |  |
| BL | 2017 | 0.23(31.19)f | 0.19(31.5)g | 0.15(31.75)h | 0.1(31.91)i | 0.33(30.27)e | 0.44(28.98)ab | 0.45(28.87)a | 0.44(29.29)ab | 0.43(29.89)bc | 0.43(30.52)cd | 0.42(31.07)d |  |
| BL | 2018 | 0.45(41.41)e | 0.07(46.27)g | 0(46.39)h | 0.09(46.23)f | 0.46(41.22)d | 0.63(36.17)a | 0.63(36.21)a | 0.63(36.28)a | 0.62(36.75)a | 0.61(37.49)b | 0.61(38.38)c |  |
| BL | 2020 | 0.22(43.85)e | -0.04(45.06)g | -0.07(45.11)g | -0.05(45.08)g | 0.19(44.24)f | 0.4(41.96)d | 0.38(42.16)d | 0.44(41.55)c | 0.48(41.48)b | 0.51(41.6)ab | 0.52(43.02)a |  |
| BL | 2016-2017 | 0.28(28.85)f | 0.14(29.8)g | 0.1(29.92)h | 0.05(30.03)i | 0.33(28.36)e | 0.44(27.21)bc | 0.41(27.52)d | 0.45(27.16)a | 0.44(27.65)ab | 0.44(28)abc | 0.43(28.61)c |  |
| BL | 2016-2018 | 0.39(36.74)f | 0.1(39.69)g | 0.05(39.82)h | 0.03(39.85)i | 0.4(36.5)e | 0.57(32.93)b | 0.51(34.19)d | 0.56(33.16)c | 0.58(32.59)a | 0.58(32.68)a | 0.58(32.91)a |  |
| BL | 2016-2020 | 0.37(37.86)g | 0.08(40.57)h | 0.03(40.69)i | 0.02(40.7)j | 0.38(37.66)f | 0.55(34.07)c | 0.49(35.55)e | 0.53(34.48)d | 0.57(33.74)b | 0.57(33.66)a | 0.58(33.78)a |  |
| DP | 2013 | 0.29(28.67)g | 0.43(27.12)d | 0.06(29.97)h | 0.34(28.24)f | 0.51(25.88)a | 0.35(28.48)f | 0.37(28.22)e | 0.42(28)d | 0.46(27.94)c | 0.47(28.08)bc | 0.48(28.44)b |  |
| DP | 2014 | 0.38(31.72)f | 0.42(31.21)e | -0.06(34.37)g | 0.38(31.84)f | 0.56(28.38)a | 0.46(30.99)d | 0.48(30.51)cd | 0.49(30.85)c | 0.51(31.05)b | 0.51(31.32)b | 0.52(31.66)b |  |
| DP | 2015 | 0.27(40.62)f | 0.43(38.13)b | -0.04(42.23)g | 0.33(39.92)e | 0.49(36.83)a | 0.34(39.99)e | 0.38(39.66)d | 0.4(39.96)c | 0.43(40.08)b | 0.43(40.38)b | 0.44(40.81)b |  |
| DP | 2016 | 0.34(47.48)e | 0.39(46.5)d | -0.08(50.63)g | 0.33(47.79)f | 0.51(43.54)a | 0.41(46.75)d | 0.43(46.32)c | 0.45(46.31)b | 0.47(47)b | 0.45(48.17)b | 0.45(48.6)b |  |
| DP | 2013-2014 | 0.36(27.09)f | 0.46(25.8)d | -0.07(29)g | 0.38(26.74)e | 0.57(23.82)a | 0.49(25.51)c | 0.48(25.46)c | 0.53(25.02)b | 0.56(25)a | 0.56(25.12)a | 0.57(25.31)a |  |
| DP | 2013-2015 | 0.28(29.26)e | 0.46(27.04)c | 0.03(30.46)f | 0.41(27.85)d | 0.54(25.68)ab | 0.47(27.22)c | 0.47(27.09)c | 0.52(26.61)b | 0.54(26.75)a | 0.55(26.84)a | 0.54(27.09)a |  |
| DP | 2013-2016 | 0.3(29.36)f | 0.46(27.27)d | 0.01(30.79)g | 0.42(27.94)e | 0.56(25.58)a | 0.49(27.15)c | 0.5(26.91)c | 0.53(26.74)b | 0.55(26.86)a | 0.56(26.85)a | 0.55(27.29)a |  |
| *Models labeled with the same letter are not significantly different (*P*-value =0.05);Numbers in parenthesis are root-mean square error of prediction accuracy.  Adjustments: ALL_M: IWB12603 IWB12603(Qyr.wpg-1B.1), KASP(Lr68), Xpsp3000(Yr10), and KASP(Yr17) combined; GWAS_B: genome-wide association assisted genomic selection (GWAS-GS) with Bonferonni significant markers; GWAS_5: GWAS-GS with the top 5 significant markers; GWAS_10: GWAS-GS with the top 10 significant markers; GWAS_25: GWAS-GS with the top 25 significant markers; GWAS_50: GWAS-GS with the top 50 significant markers; GWAS_100: GWAS-GS with the top 100 significant markers. | | | | | | | | | | | | | |

| **Table S6.** Comparison of genomic selection models accuracy, root-mean square error, and pairwise comparisons for stripe rust infection type for Pacific Northwest winter wheat diversity panel (DP) lines and breeding lines (BL) phenotyped from 2013-2020 in Central Ferry, Lind, and Pullman, WA using validation sets. | | | | | | | | | | | | |
| --- | --- | --- | --- | --- | --- | --- | --- | --- | --- | --- | --- | --- |
| Validation Set | rrBLUP | IWB12603 | *Lr68* | *Yr10* | *Yr17* | All_M | GWAS_B | GWAS_5 | GWAS_10 | GWAS_25 | GWAS_50 | GWAS_100 |
| 2013 to 2014 | 0.56(2.52)d* | 0.57(2.52)a | 0.56(2.52)f | 0.57(2.52)b | 0.57(2.52)c | 0.56(2.52)e | 0.55(2.55)g | 0.54(2.56)j | 0.54(2.56)k | 0.53(2.58)l | 0.54(2.56)h | 0.54(2.57)i |
| 2013-2014 to 2015 | 0.65(2.43)d | 0.65(2.43)f | 0.66(2.43)b | 0.65(2.43)c | 0.65(2.43)e | 0.66(2.42)a | 0.65(2.44)i | 0.65(2.44)h | 0.65(2.43)g | 0.62(2.49)j | 0.62(2.49)k | 0.61(2.53)l |
| 2013-2015 to 2016 | 0.53(3.69)c | 0.53(3.68)b | 0.53(3.7)d | 0.53(3.68)a | 0.52(3.7)f | 0.52(3.7)e | 0.52(3.69)g | 0.5(3.71)h | 0.49(3.73)i | 0.42(3.83)l | 0.44(3.79)k | 0.45(3.77)j |
| 2013-2016 to 2017 | 0.37(3.98)f | 0.38(3.96)d | 0.37(3.96)g | 0.36(3.98)h | 0.37(3.97)e | 0.38(3.93)c | 0.39(3.92)b | 0.39(3.92)a | 0.35(4.02)i | 0.29(4.11)j | 0.25(4.24)k | 0.25(4.28)l |
| 2013-2017 to 2018 | 0.27(5.55)j | 0.31(5.48)e | 0.28(5.53)g | 0.28(5.53)h | 0.28(5.53)i | 0.31(5.49)f | 0.32(5.46)b | 0.33(5.45)a | 0.32(5.48)c | 0.31(5.5)d | 0.27(5.61)k | 0.23(5.74)l |
| 2013-2018 to 2020 | 0.51(5.59)k | 0.52(5.57)f | 0.52(5.58)h | 0.51(5.58)j | 0.52(5.58)g | 0.52(5.56)e | 0.55(5.46)a | 0.53(5.53)d | 0.54(5.5)b | 0.54(5.49)c | 0.5(5.63)l | 0.51(5.67)i |
| *Models labeled with the same letter are not significantly different (*P*-value =0.05); Numbers in parenthesis are root-mean square error of prediction accuracy.  Adjustments: ALL_M: IWB12603(Qyr.wpg-1B.1), KASP(Lr68), Xpsp3000(Yr10), and KASP(Yr17) combined; GWAS_B: genome-wide association assisted genomic selection (GWAS-GS) with Bonferonni significant markers; GWAS_5: GWAS-GS with the top 5 significant markers; GWAS_10: GWAS-GS with the top 10 significant markers; GWAS_25: GWAS-GS with the top 25 significant markers; GWAS_50: GWAS-GS with the top 50 significant markers; and GWAS_100: GWAS-GS with the top 100 significant markers. | | | | | | | | | | | | |

| **Table S7.** Comparison of genomic selection models accuracy, root-mean square error, and pairwise comparisons for stripe rust disease severity for Pacific Northwest winter wheat diversity panel (DP) lines and breeding lines (BL) phenotyped from 2013-2020 in Central Ferry, Lind, and Pullman, WA using validation sets. | | | | | | | | | | | | |
| --- | --- | --- | --- | --- | --- | --- | --- | --- | --- | --- | --- | --- |
| Validation Set | rrBLUP | IWB12603 | *Lr68* | *Yr10* | *Yr17* | All_M | GWAS_B | GWAS_5 | GWAS_10 | GWAS_25 | GWAS_50 | GWAS_100 |
| 2013 to 2014 | 0.72(23.95)e* | 0.72(23.9)b | 0.72(23.98)f | 0.72(23.92)c | 0.72(23.95)d | 0.72(23.9)a | 0.71(24.19)h | 0.71(24.13)g | 0.7(24.4)i | 0.69(24.94)l | 0.69(24.86)k | 0.69(24.82)j |
| 2013-2014 to 2015 | 0.7(36.65)h | 0.7(36.65)e | 0.71(36.6)d | 0.7(36.64)f | 0.7(36.65)g | 0.71(36.6)a | 0.71(36.62)b | 0.71(36.57)c | 0.7(36.84)i | 0.69(37.02)j | 0.69(37.11)k | 0.68(37.24)l |
| 2013-2015 to 2016 | 0.64(35.11)b | 0.64(35.02)a | 0.63(35.45)e | 0.64(35.13)c | 0.63(35.3)d | 0.63(35.48)f | 0.59(36.44)g | 0.58(36.8)h | 0.57(36.92)i | 0.52(38.2)l | 0.54(37.7)k | 0.55(37.47)j |
| 2013-2016 to 2017 | 0.42(30.8)d | 0.42(30.7)a | 0.42(30.63)b | 0.41(30.87)g | 0.42(30.74)e | 0.42(30.53)c | 0.38(31.42)j | 0.38(31.42)k | 0.4(31.23)h | 0.41(31.36)f | 0.38(31.85)i | 0.37(32.4)l |
| 2013-2017 to 2018 | 0.32(44.02)l | 0.34(43.59)e | 0.33(43.85)i | 0.33(43.81)g | 0.33(43.84)j | 0.34(43.64)f | 0.38(43.07)a | 0.33(43.87)h | 0.37(43.2)b | 0.35(43.42)c | 0.35(43.52)d | 0.32(44.15)k |
| 2013-2018 to 2020 | 0.47(40.46)j | 0.44(40.45)l | 0.46(40.47)k | 0.47(40.44)i | 0.47(40.45)h | 0.51(40.43)a | 0.5(40.28)b | 0.47(39.66)f | 0.5(39.84)c | 0.47(40.67)e | 0.47(40.49)g | 0.48(39.44)d |
| *Models labeled with the same letter are not significantly different (*P*-value =0.05); Numbers in parenthesis are root-mean square error of prediction accuracy.  Adjustments: ALL_M: IWB12603(Qyr.wpg-1B.1), KASP(Lr68), Xpsp3000(Yr10), and KASP(Yr17) combined; GWAS_B: genome-wide association assisted genomic selection (GWAS-GS) with Bonferonni significant markers; GWAS_5: GWAS-GS with the top 5 significant markers; GWAS_10: GWAS-GS with the top 10 significant markers; GWAS_25: GWAS-GS with the top 25 significant markers; GWAS_50: GWAS-GS with the top 50 significant markers; and GWAS_100: GWAS-GS with the top 100 significant markers. | | | | | | | | | | | | |

| **Table S8.** Comparison of marker-assisted selection models accuracy, root-mean square error, and pairwise comparisons for stripe rust infection type for Pacific Northwest winter wheat diversity panel (DP) lines and breeding lines (BL) phenotyped from 2013-2020 in Central Ferry, Lind, and Pullman, WA using validation sets. | | | | | | | | | | | | |
| --- | --- | --- | --- | --- | --- | --- | --- | --- | --- | --- | --- | --- |
| Validation Set | IWB12603 | *Lr68* | *Yr10* | *Yr17* | All_M | GWAS_B | GWAS_5 | GWAS_10 | GWAS_25 | GWAS_50 | GWAS_100 |  |
| 2013 to 2014 | 0.21(2.98)i* | 0.24(2.96)h | 0.01(3.04)k | 0.14(3.02)j | 0.29(2.92)g | 0.32(2.89)f | 0.33(2.89)e | 0.41(2.79)d | 0.47(2.7)c | 0.5(2.65)b | 0.56(2.6)a |  |
| 2013-2014 to 2015 | 0.17(3)i | 0.42(2.83)e | 0.08(3.03)k | 0.33(2.92)f | 0.44(2.8)d | 0.13(3.03)j | 0.24(2.97)h | 0.26(2.96)g | 0.56(2.61)c | 0.57(2.6)b | 0.62(2.54)a |  |
| 2013-2015 to 2016 | 0.33(4.12)c | 0.12(4.25)h | -0.03(4.25)k | 0.05(4.28)i | 0.23(4.16)e | 0.2(4.19)f | 0.2(4.19)g | 0.3(4.06)d | 0.35(3.95)b | 0.37(3.93)a | 0.04(28.38)j |  |
| 2013-2016 to 2017 | 0.21(4.18)f | 0.15(4.28)g | -0.15(4.31)j | 0.06(4.29)i | 0.24(4.15)e | 0.38(4.03)a | 0.38(4.03)a | 0.3(4.13)b | 0.26(4.17)c | 0.24(4.26)d | 0.12(7.48)h |  |
| 2013-2017 to 2018 | 0.45(5.55)b | 0.07(5.85)i | -0.01(5.85)j | -0.09(5.87)k | 0.43(5.55)d | 0.43(5.41)c | 0.47(5.45)a | 0.39(5.45)e | 0.3(5.59)f | 0.25(5.71)g | 0.23(5.99)h |  |
| 2013-2018 to 2020 | 0.25(6.01)h | 0.08(6.19)i | 0.07(6.21)j | -0.1(6.22)k | 0.27(5.99)g | 0.51(5.53)a | 0.47(5.71)d | 0.46(5.65)e | 0.5(5.54)b | 0.5(5.61)c | 0.42(5.74)f |  |
| *Models labeled with the same letter are not significantly different (*P*-value =0.05); Numbers in parenthesis are root-mean square error of prediction accuracy.  Adjustments: ALL_M: IWB12603(Qyr.wpg-1B.1), KASP(Lr68), Xpsp3000(Yr10), and KASP(Yr17) combined; GWAS_B: genome-wide association assisted genomic selection (GWAS-GS) with Bonferonni significant markers; GWAS_5: GWAS-GS with the top 5 significant markers; GWAS_10: GWAS-GS with the top 10 significant markers; GWAS_25: GWAS-GS with the top 25 significant markers; GWAS_50: GWAS-GS with the top 50 significant markers; and GWAS_100: GWAS-GS with the top 100 significant markers. | | | | | | | | | | | | |

| **Table S9.** Comparison of marker-assisted selection models accuracy, root-mean square error, and pairwise comparisons for stripe rust disease severity for Pacific Northwest winter wheat diversity panel (DP) lines and breeding lines (BL) phenotyped from 2013-2020 in Central Ferry, Lind, and Pullman, WA using validation sets. | | | | | | | | | | | | |
| --- | --- | --- | --- | --- | --- | --- | --- | --- | --- | --- | --- | --- |
| Validation Set | IWB12603 | *Lr68* | *Yr10* | *Yr17* | All_M | GWAS_B | GWAS_5 | GWAS_10 | GWAS_25 | GWAS_50 | GWAS_100 |  |
| 2013 to 2014 | 0.38(31.98)i* | 0.42(31.18)h | -0.01(34.37)k | 0.37(31.91)j | 0.56(28.71)f | 0.56(28.4)e | 0.55(28.56)g | 0.6(27.35)d | 0.63(26.63)c | 0.65(26.23)b | 0.68(25.88)a |  |
| 2013-2014 to 2015 | 0.27(45.48)j | 0.43(43.57)h | 0.02(46.9)k | 0.33(44.91)i | 0.5(42.19)g | 0.55(40.87)e | 0.51(41.79)f | 0.59(40.01)d | 0.59(39.89)c | 0.64(38.53)b | 0.68(37.09)a |  |
| 2013-2015 to 2016 | 0.41(41.53)e | 0.15(45.19)h | -0.05(44.77)k | 0.12(45.15)i | 0.38(41.7)f | 0.48(39.41)b | 0.26(43.97)g | 0.47(39.5)c | 0.46(39.7)d | 0.5(38.8)a | 0.07(381.71)j |  |
| 2013-2016 to 2017 | 0.23(32.06)e | 0.19(32.95)f | -0.15(33.43)j | 0.1(33.23)i | 0.29(31.73)d | 0.11(34.69)h | 0.11(34.69)h | 0.3(32.1)c | 0.34(32.81)b | 0.34(32.36)a | 0.13(63.83)g |  |
| 2013-2017 to 2018 | 0.45(43.52)a | 0.07(46.61)i | 0.02(46.59)j | -0.09(46.97)k | 0.42(43.6)c | 0.43(42.72)b | 0.08(46.8)h | 0.36(44.44)e | 0.36(43.22)d | 0.35(43.51)f | 0.18(51.27)g |  |
| 2013-2018 to 2020 | 0.23(43.99)g | 0.02(45.04)i | 0.02(45.1)k | 0.02(45.03)j | 0.22(43.96)h | 0.41(41.37)e | 0.41(41.57)d | 0.47(40.83)a | 0.41(41.25)f | 0.45(40.69)b | 0.43(40.65)c |  |
| *Models labeled with the same letter are not significantly different (*P*-value =0.05): Numbers in parenthesis are root-mean square error of prediction accuracy.  Adjustments: ALL_M: IWB12603(Qyr.wpg-1B.1), KASP(Lr68), Xpsp3000(Yr10), and KASP(Yr17) combined; GWAS_B: genome-wide association assisted genomic selection (GWAS-GS) with Bonferonni significant markers; GWAS_5: GWAS-GS with the top 5 significant markers; GWAS_10: GWAS-GS with the top 10 significant markers; GWAS_25: GWAS-GS with the top 25 significant markers; GWAS_50: GWAS-GS with the top 50 significant markers; and GWAS_100: GWAS-GS with the top 100 significant markers. | | | | | | | | | | | | |


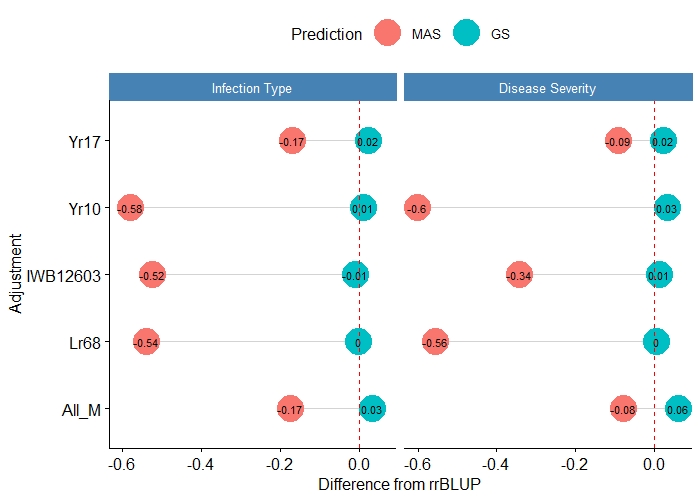


**Figure S1 |** Difference in prediction accuracy from the base rrBLUP model for major markers in genomic selection (GS) and marker-assisted selection (MAS) in the breeding lines in 2016. Adjustments: ALL_M: IWB12603(Qyr.wpg-1B.1), KASP(Lr68), Xpsp3000(Yr10), and KASP(Yr17) combined.


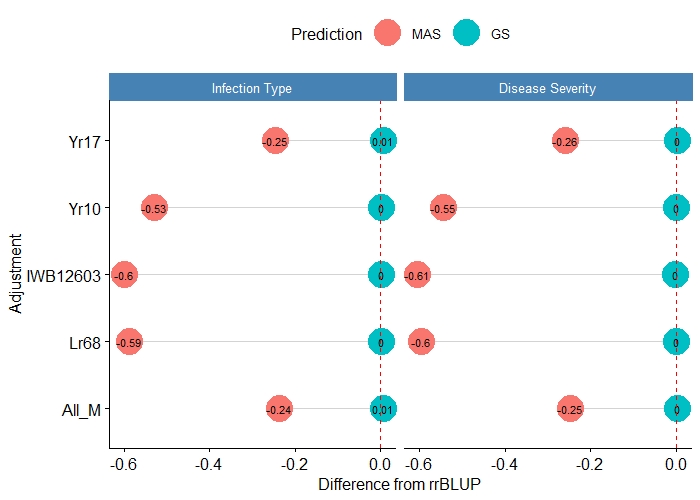


**Figure S2 |** Difference in prediction accuracy from the base rrBLUP model for major markers genomic selection (GS) and marker-assisted selection (MAS) in the breeding lines across 2016-2020. Adjustments: ALL_M: IWB12603(Qyr.wpg-1B.1), KASP(Lr68), Xpsp3000(Yr10), and KASP(Yr17) combined.


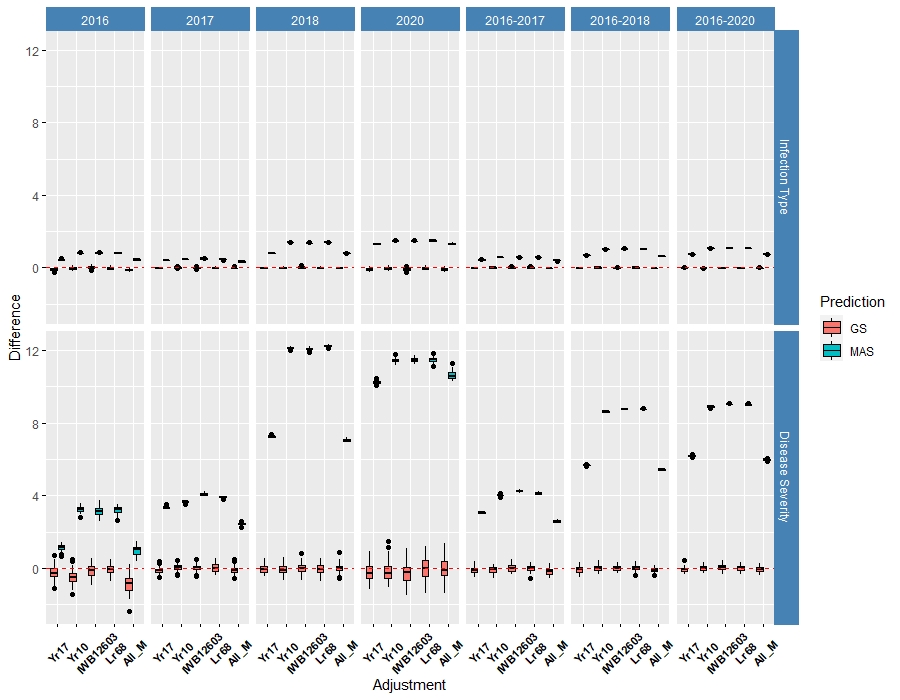


**Figure S3 |** Difference in root mean square error for predictions from the base rrBLUP model for major markers in genomic selection (GS) and marker-assisted selection (MAS) using cross-validations in the breeding lines phenotyped from 2016 to 2020. Adjustments: ALL_M: IWB12603(Qyr.wpg-1B.1), KASP(Lr68), Xpsp3000(Yr10), and KASP(Yr17) combined.


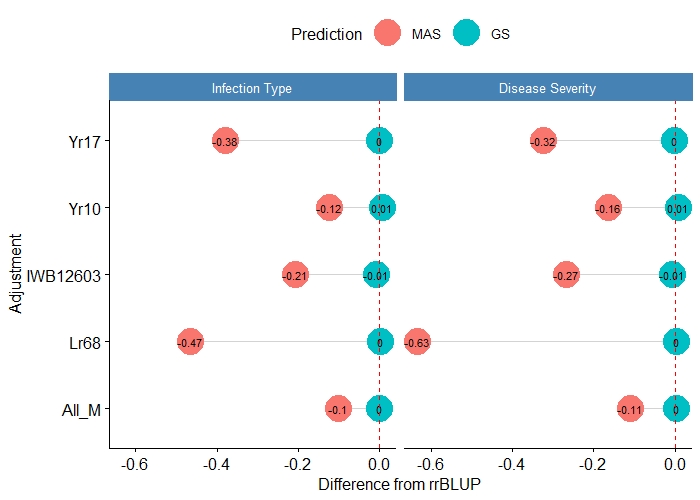


**Figure S4 |** Difference in prediction accuracy from the base rrBLUP model for major markers in genomic selection (GS) and marker-assisted selection (MAS) in the diversity panel lines in 2015. Adjustments: ALL_M: IWB12603(Qyr.wpg-1B.1), KASP(Lr68), Xpsp3000(Yr10), and KASP(Yr17) combined.


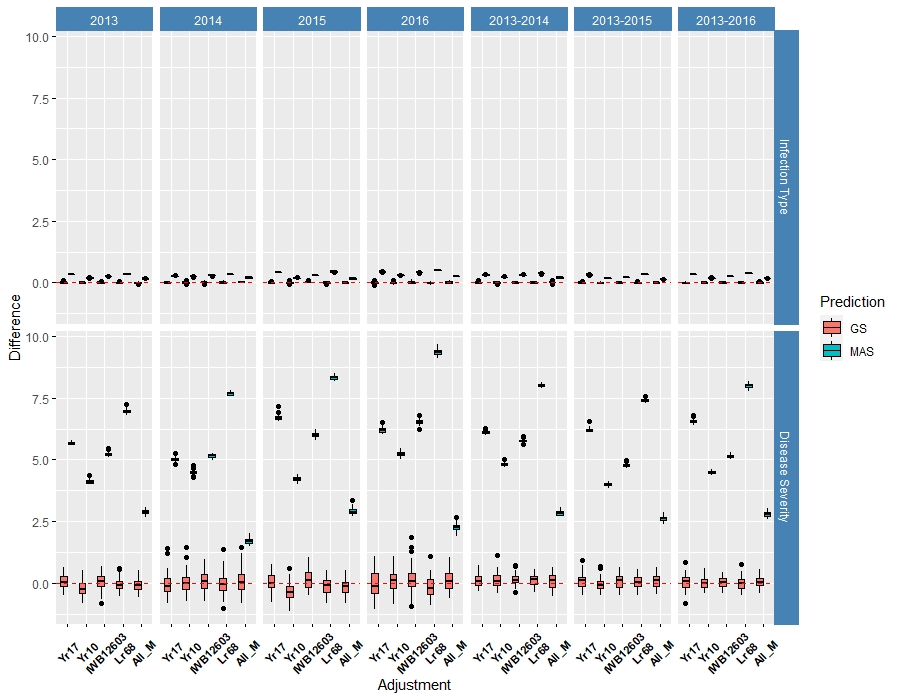


**Figure S5 |** Difference in root mean square error for predictions from the base rrBLUP model for major markers in genomic selection (GS) and marker-assisted selection (MAS) using cross-validations in the breeding lines phenotyped from 2016 to 2020. Adjustments: ALL_M: IWB12603(Qyr.wpg-1B.1), KASP(Lr68), Xpsp3000(Yr10), and KASP(Yr17) combined.


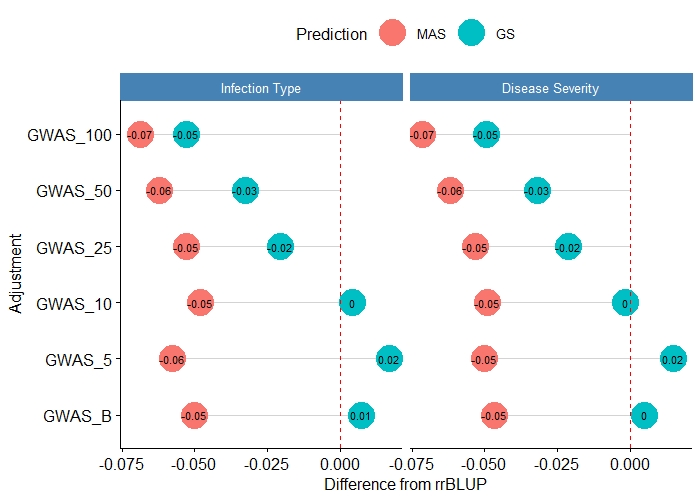


**Figure S6 |** Difference in prediction accuracy from the base rrBLUP model for de novo GWAS markers in genomic selection (GS) and marker-assisted selection (MAS) in the breeding lines in 2018. Adjustments: GWAS_B: genome-wide association assisted genomic selection (GWAS-GS) with Bonferonni significant markers; GWAS_5: GWAS-GS with the top 5 significant markers; GWAS_10: GWAS-GS with the top 10 significant markers; GWAS_25: GWAS-GS with the top 25 significant markers; GWAS_50: GWAS-GS with the top 50 significant markers; and GWAS_100: GWAS-GS with the top 100 significant markers.


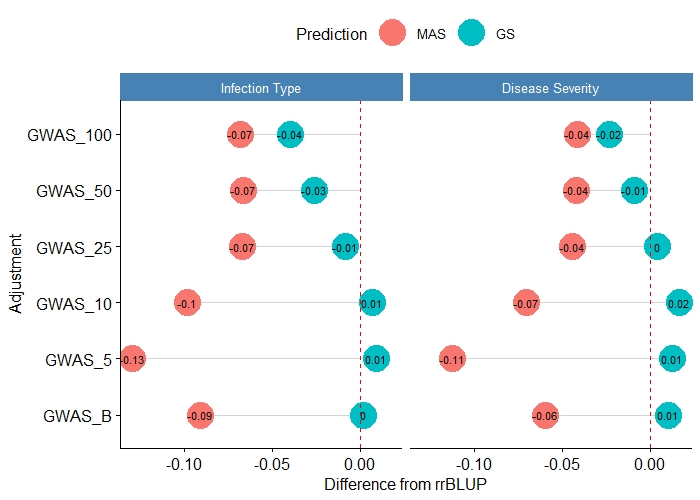


**Figure S7.** Difference in prediction accuracy from the base rrBLUP model for de novo GWAS markers in genomic selection (GS) and marker-assisted selection (MAS) in the breeding lines across 2016-2018. Adjustments: GWAS_B: genome-wide association assisted genomic selection (GWAS-GS) with Bonferonni significant markers; GWAS_5: GWAS-GS with the top 5 significant markers; GWAS_10: GWAS-GS with the top 10 significant markers; GWAS_25: GWAS-GS with the top 25 significant markers; GWAS_50: GWAS-GS with the top 50 significant markers; GWAS_100: GWAS-GS with the top 100 significant markers.


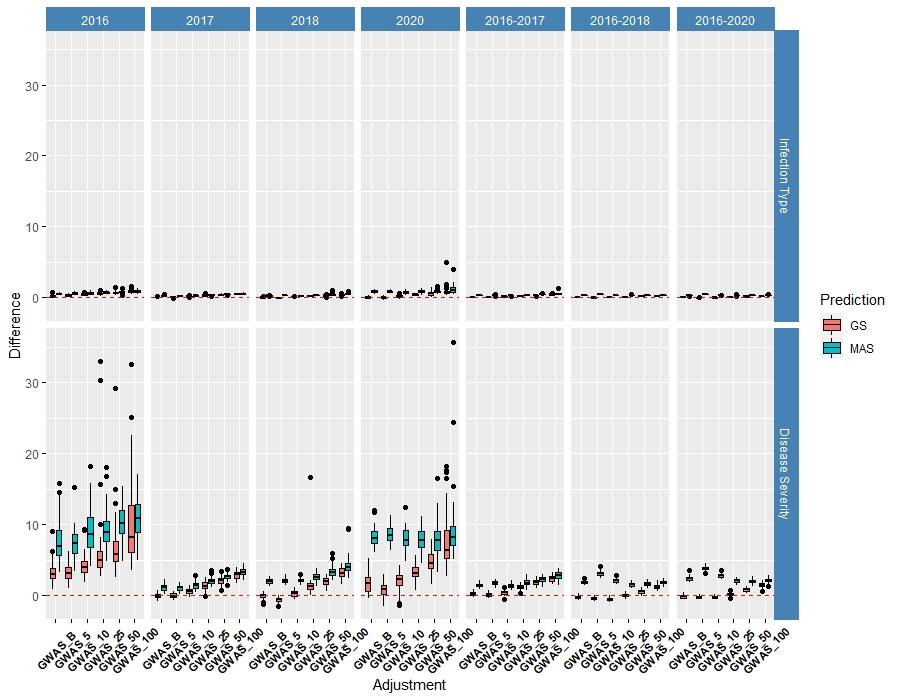


**Figure S8 |** Difference in root mean square error for predictions from the base rrBLUP model for de novo GWAS markers in genomic selection (GS) and marker-assisted selection (MAS) using cross-validations in the breeding lines phenotyped from 2016 to 2020. Adjustments: GWAS_B: genome-wide association assisted genomic selection (GWAS-GS) with Bonferonni significant markers; GWAS_5: GWAS-GS with the top 5 significant markers; GWAS_10: GWAS-GS with the top 10 significant markers; GWAS_25: GWAS-GS with the top 25 significant markers; GWAS_50: GWAS-GS with the top 50 significant markers; and GWAS_100: GWAS-GS with the top 100 significant markers.


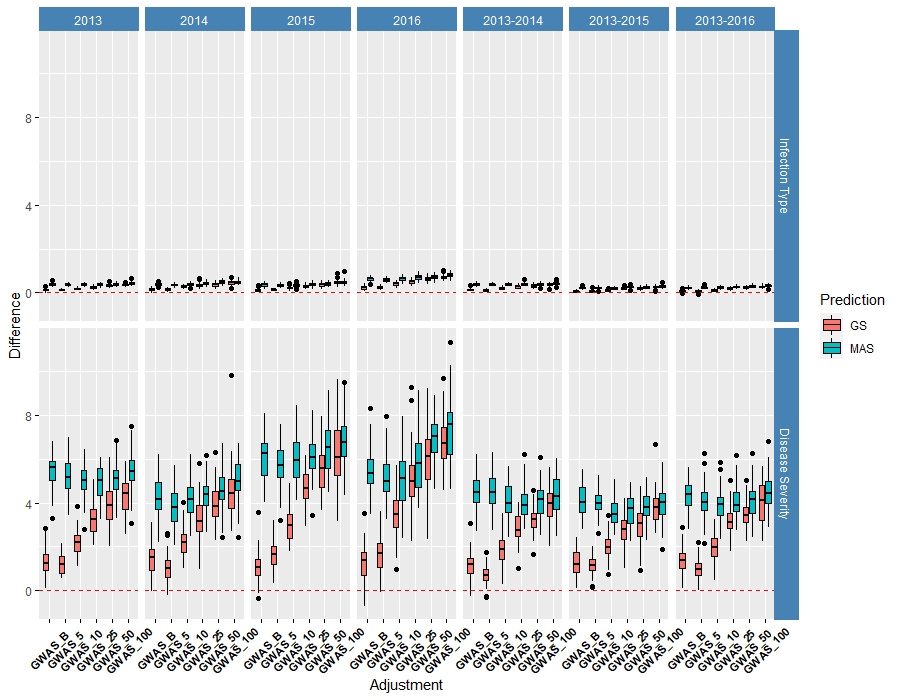


**Figure S9 |** Difference in root mean square error for predictions from the base rrBLUP model for de novo GWAS markers in genomic selection (GS) and marker-assisted selection (MAS) using cross-validations in the diversity panel lines phenotyped from 2013 to 2016. Adjustments: GWAS_B: genome-wide association assisted genomic selection (GWAS-GS) with Bonferonni significant markers; GWAS_5: GWAS-GS with the top 5 significant markers; GWAS_10: GWAS-GS with the top 10 significant markers; GWAS_25: GWAS-GS with the top 25 significant markers; GWAS_50: GWAS-GS with the top 50 significant markers; and GWAS_100: GWAS-GS with the top 100 significant markers.


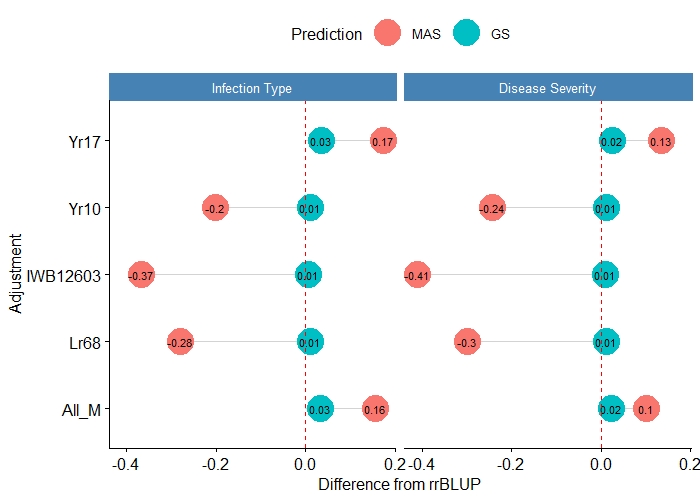


**Figure S10 |** Difference in prediction accuracy from the base rrBLUP model for major markers in genomic selection (GS) and marker-assisted selection (MAS)in the validation set using the years 2013-2017 to predict 2018. Adjustments: ALL_M: IWB12603(Qyr.wpg-1B.1), KASP(Lr68), Xpsp3000(Yr10), and KASP(Yr17) combined.


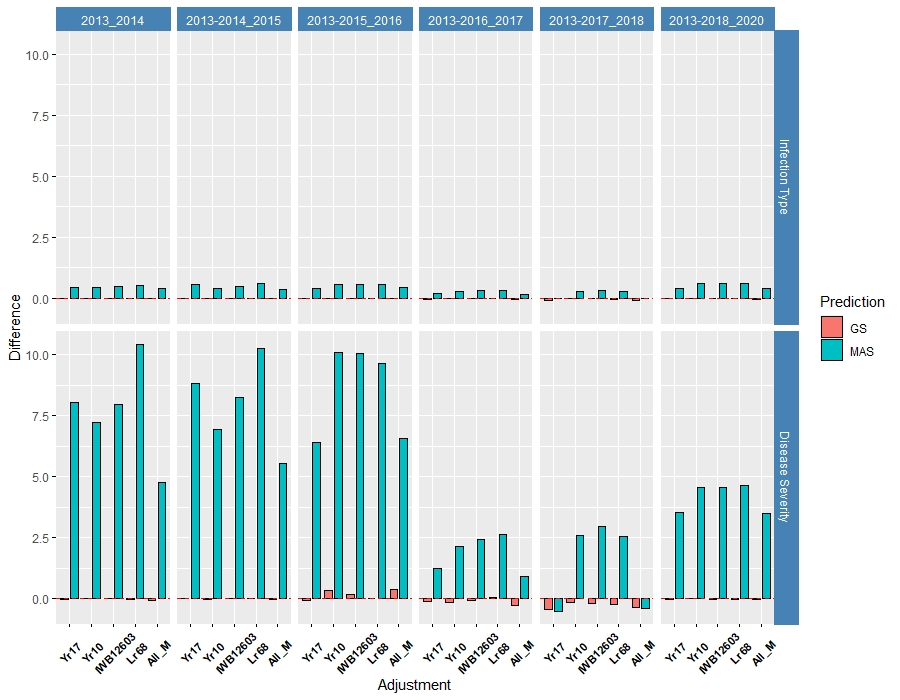


**Figure S11 |** Difference in root mean square error for predictions from the base rrBLUP model for major markers in genomic selection (GS) and marker-assisted selection (MAS) in the validation set using the diversity panel and breeding lines phenotyped from 2013 predict 2020. Adjustments: ALL_M: IWB12603(Qyr.wpg-1B.1), KASP(Lr68), Xpsp3000(Yr10), and KASP(Yr17) combined.


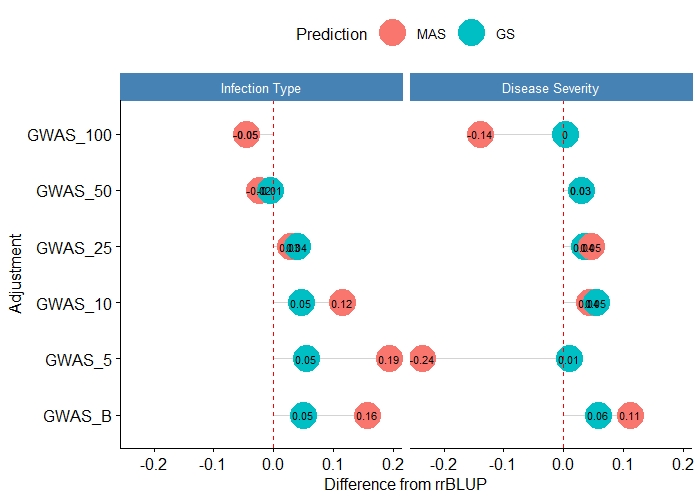


**Figure S12 |** Difference in prediction accuracy from the base rrBLUP Model for de novo GWAS markers in genomic selection (GS) and marker-assisted selection (MAS)in the validation set using the years 2013-2017 to predict 2018. Adjustments: GWAS_B: genome-wide association assisted genomic selection (GWAS-GS) with Bonferonni significant markers; GWAS_5: GWAS-GS with the top 5 significant markers; GWAS_10: GWAS-GS with the top 10 significant markers; GWAS_25: GWAS-GS with the top 25 significant markers; GWAS_50: GWAS-GS with the top 50 significant markers; and GWAS_100: GWAS-GS with the top 100 significant markers.


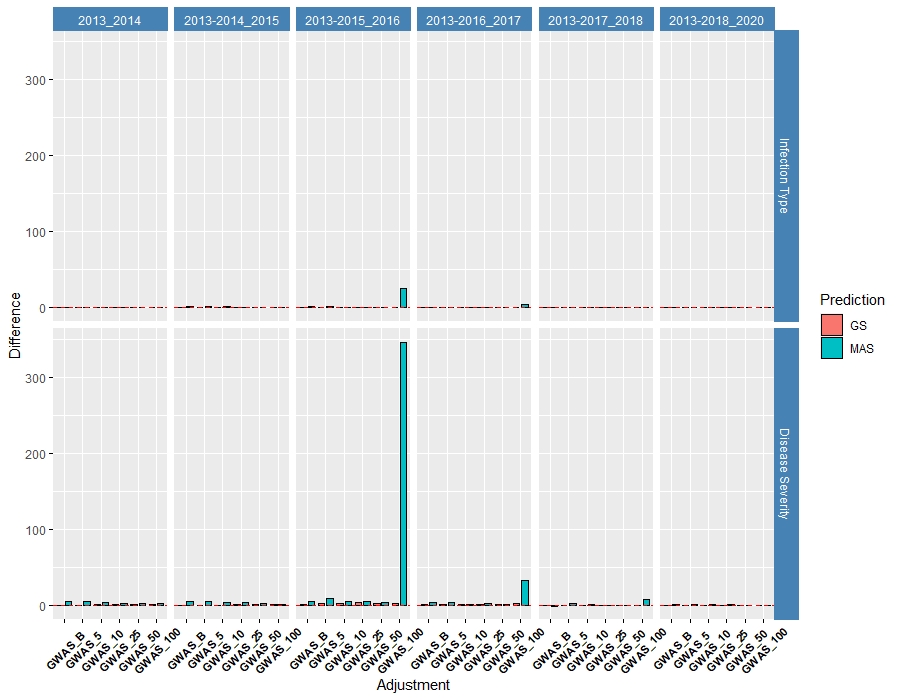


**Figure S13 |** Difference in root mean square error for predictions from the base rrBLUP model for de novo GWAS markers in genomic selection (GS) and marker-assisted selection (MAS) in the validation set using the diversity panel and breeding lines phenotyped from 2013 predict 2020. Adjustments: GWAS_B: genome-wide association assisted genomic selection (GWAS-GS) with Bonferonni significant markers; GWAS_5: GWAS-GS with the top 5 significant markers; GWAS_10: GWAS-GS with the top 10 significant markers; GWAS_25: GWAS-GS with the top 25 significant markers; GWAS_50: GWAS-GS with the top 50 significant markers; and GWAS_100: GWAS-GS with the top 100 significant markers.
